# Disease-associated microglial activation prevents photoreceptor degeneration by suppressing the accumulation of cell debris and neutrophils in degenerating rat retinas

**DOI:** 10.7150/thno.67954

**Published:** 2022-03-06

**Authors:** Juncai He, Yan Fu, Lingling Ge, Jiaman Dai, Yajie Fang, Yijian Li, Xianliang Gu, Zui Tao, Ting Zou, Minghui Li, Yong Liu, Haiwei Xu, Zheng Qin Yin

**Affiliations:** 1 Southwest Hospital/Southwest Eye Hospital, Third Military Medical University (Amy Medical University), Chongqing, 400038, P.R. China; 2 Key Lab of Visual Damage and Regeneration & Restoration of Chongqing, Chongqing, 400038, P.R. China; 3 No. 927 Hospital, Joint Logistics Support Force of Chinese PLA, Puer 665000, Yunnan, China; 4 The General Hospital of Western Theater Command, Chengdu 610083, China

**Keywords:** microglia, disease-associated microglia, photoreceptor degeneration, neutrophil, photoreceptor

## Abstract

Retinitis pigmentosa initially presents as night blindness owing to defects in rods, and the secondary degeneration of cones ultimately leads to blindness. Previous studies have identified active roles of microglia in the pathogenesis of photoreceptor degeneration in RP. However, the contribution of microglia to photoreceptor degeneration remains controversial, partly due to limited knowledge of microglial phenotypes during RP.

**Rationale:** In this study, we investigated the pathways of microglial activation and its contribution to photoreceptor degeneration in RP.

**Methods:** A classic RP model, Royal College of Surgeons rat, was used to explore the process of microglial activation during the development of RP. An inhibitor of colony-stimulating factor 1 receptor (PLX3397) was fed to RCS rats for sustained ablation of microglia. Immunohistochemistry, flow cytometry, RT-qPCR, electroretinography and RNA-Seq were used to investigate the mechanisms by which activated microglia influenced photoreceptor degeneration.

**Results:** Microglia were gradually activated to disease-associated microglia in the photoreceptor layers of RCS rats. Sustained treatment with PLX3397 ablated most of the disease-associated microglia and aggravated photoreceptor degeneration, including the secondary degeneration of cones, by downregulating the expression of genes associated with photoreceptor function and components and exacerbating the impairment of photoreceptor cell function. Disease-associated microglial activation promoted microglia to engulf apoptotic photoreceptor cell debris and suppressed the increase of infiltrated neutrophils by increasing engulfment and inhibiting CXCL1 secretion by Müller cells, which provided a healthier microenvironment for photoreceptor survival.

**Conclusions:** Our data highlight a key role of disease-associated microglia activation in the suppression of rod and cone degeneration, which reduces secondary damage caused by the accumulation of dead cells and infiltrated neutrophils in the degenerating retina.

## Introduction

Retinitis pigmentosa (RP) refers to a family of retinal disorders that cause blindness, typically attributed to hereditary factors. These disorders are characterized by a common pathological process—the progressive deterioration and loss of photoreceptors 1,2. In most RP models, genetic mutations lead to primary rod-specific damage and cause night blindness, almost invariably followed by a secondary degeneration of the cones. When most rods have been lost, the cone photoreceptors slowly degenerate in a common and characteristic pattern, causing the loss of high visual acuity and color vision 3,4. However, the mechanisms that regulate the loss of rods and cones are not completely understood, resulting in a lack of effective treatments to limit progressive photoreceptor degeneration in RP.

Adult retinal microglia are highly specialized, embryonically derived, long-lived tissue macrophages, and are the predominant type of immune cell in the retina; they primarily reside in the outer and inner plexiform layers (OPL and IPL) 5. Microglial reactivity is a key feature of RP, and is accompanied by microglial invasion to the photoreceptor layers, proliferation and activation of a less-ramified, more-amoeboid morphology 6-8. Importantly, activated microglia are considered a central player in the degeneration of photoreceptors, including rods and cones 9. However, the protective versus pathogenic roles of microglia in photoreceptor loss have been intensely debated. The death of degenerating rods during inherited RP is exacerbated by microglial secretion of cytokines, which induce neurotoxicity and apoptosis signaling molecules as well as microglial phagocytosis of non-apoptotic photoreceptors 10,11. In contrast, microglia have also been found to inhibit photoreceptor death by removing potentially damaging cell debris and regulating immune cell infiltration in acute and chronic models of photoreceptor degeneration 12-14. Furthermore, photoreceptor degeneration remains unaffected upon microglia depletion by a colony-stimulating factor 1 receptor (CSF1R) inhibitor or by Cx3cr1 knockout in prion-infected 15 and Mertk-mutated retinas 16, implying that microglia are not required for photoreceptor degeneration. The role and function of microglia in photoreceptor degeneration thus remain controversial, probably due to the existence of multiple phenotypes of microglia at different stages of disease progression. However, the phenotypes of activated microglia and the corresponding pathways of microglial activation during RP are not well understood.

Microglial phenotypes are classically identified according to morphology or M1/M2 patterns (borrowed from the now-defunct classification of macrophages), yet this system is unable to specify a particular response state or type of activity in a given neurodegenerative disease 17, including RP 18. Recently, single-cell RNA analysis has identified disease-associated microglia (DAM), a subset of microglia with a conserved transcriptional and functional signature across neurodegenerative models 19-21. Intriguingly, the transcriptional signature of DAM includes the upregulation of genes characteristically expressed by classic M1 and M2 macrophages, as well as modules for lipid metabolism and phagocytic pathways (e.g., Axl, Clec7a, Cst7, Spp1, Tyrobp, Lgals3, Apoe, and Trem2), and downregulation of genes expressed by homeostatic microglia (e.g., Cx3cr1, Tmem119 and P2ry12/P2ry13) 19-21. However, whether microglia are activated to the DAM phenotype in the RP (as the retina is considered a discrete central nervous system (CNS) region) remains to be determined. Therefore, we further investigated whether microglia were gradually activated to DAM and how DAM activation contributed to the photoreceptor degeneration.

Clarifying the role of microglia in photoreceptor degeneration requires the prolonged depletion of microglia. The survival of microglia has previously been shown to be critically dependent on CSF1R signaling. Recently, CSF1R inhibitors have been used to rapidly eliminate most microglia in the CNS to elucidate the role of microglia in neurodegenerative diseases 16,22,23. PLX3397, a CSF1R inhibitor, is orally bioavailable and capable of penetrating the brain and retina, providing a noninvasive/nonstressful approach to achieve robust brain/retina-wide microglial elimination for extended periods of time 24,25. Previous studies have found that some residual microglia survive in the diseased region in neurodegenerative models treated with a CSF1R inhibitor 22,23,26,27, providing further support for the existence of heterogeneous populations of microglia. However, the differential depletion effects of CSF1R inhibitors on microglia and the phenotype of these CSF1R inhibitor-resistant cells are not completely understood; thus further knowledge of this phenomenon may be helpful for identifying the subtypes of microglia that play a key role in pathological progression.

In the present study, we explored the pathways of microglial activation and its contribution to photoreceptor loss and dysfunction in RP. For this purpose, we used Royal College of Surgeons (RCS) rats, a well-characterized rat model of RP that carries a mutation in the Mertk gene , which first results in rod death and then cone loss 16,28,29, to explore whether microglia were activated to the DAM phenotype in degenerating retinas. To validate the effects of microglial activation on photoreceptor loss and dysfunction, RCS rats was fed PLX3397 for sustained ablation of the microglia. Thus, we determined the potential mechanisms underlying the effects of DAM activation on photoreceptor survival in RCS rats according to DAM function. Our results identified the DAM phenotype as a protective microglial subtype that suppressed photoreceptor degeneration, highlighting the potential of DAM activation as a pathogenesis-independent treatment for RP to improve visual function.

## Materials and Methods

### Ethics statements

This study was carried out by the recommendations of the Third Military Medical University Animal Care and Use Committee. The protocol was approved by the Animal Center of the Third Military Medical University.

### Animals

RCS-rdy-p^+^ (RCS rats; P15, 20, 30, 40, and 50; either sex) and RCS-rdy^+^-p^+^ rats (Control rats, P15, 20, 30, 40, and 50; either sex) were obtained from the Animal Center of the Third Military Medical University (Chongqing, China). All rats were housed in the laboratory animal facility of Southwest Hospital under a 12 h light and 12 h dark cycle with ad libitum access to food and water.

### Drug administration

Control or RCS rats were fed a PLX3397-formulated AIN-76A diet (600 p.p.m; 600 mg PLX3397 (Selleckchem, Houston, TX) per kilogram of diet) ad libitum at P15 16. The control RCS rats were fed a normal AIN-76A diet. Morphological and functional experiments were carried out in the rats after CSF1R inhibitor treatment for 5, 15, 25, and 35 days.

### Immunohistochemistry

Immunofluorescence staining of frozen tissue sections was performed as previously described 16. Briefly, the enucleated eyecups were immersed in 4% paraformaldehyde (PFA) at 4 °C for 15 min and then transferred to 30% sucrose at 4 °C overnight. The retinas were embedded in optimal cutting temperature (OCT) compound and cut into 20- or 40-µm-thick sections in the sagittal plane using a freezing microtome. Slices from a distance of 50 µm lateral to the optic nerve were selected for immunohistochemistry and analysis. Sections were permeabilized and blocked with PT1 (phosphate-buffered saline (PBS) containing 0.1% Triton X-100 and 10% donkey or goat serum) at 37 °C for 30 min and then incubated with primary antibodies (Table S1) in PT2 (PBS containing 0.03% Triton X-100 and 5% donkey or goat serum) overnight at 4 °C. After five washes with PBS, the sections were incubated with fluorophore-conjugated secondary antibodies in PT2 at 37 °C for 1 h. Nuclei were counterstained with 4',6-diamidino-2-phenylindole (DAPI; Sigma-Aldrich). CellROX Green (Life Technologies) oxidative stress reagent was used to staining reactive oxygen species (ROS), which was injected into the SRS using a 33-gauge Hamilton needle (Hamilton) and incubated for 2 h. Images of immunofluorescence staining were acquired using a confocal microscopy system (Zeiss LSM 780).

Analysis of apoptotic cells and surviving cells was performed according to a previous study 16. Total cells (DAPI) and apoptotic cells (TUNEL) in the ONL were counted in central and peripheral regions. Surviving cells were defined by DAPI-positive nuclei without TUNEL staining: number of surviving cells = number of total cells - number of apoptotic cells. These numbers were averaged for five eyes per group.

Analysis of microglia number and morphology was performed according to a previous study 16,30. Panoramic sections of the retina per group immunostained for DAPI and IBA1 were captured to quantify the numbers of microglial cells in the different retinal layers. The numbers of grid-crossing points and processes per individual cells were used to analyze the morphology of the microglia.

Images were acquired with a confocal microscope at a size of 135 × 135 µm with 0.2-µm z-steps for analysis of microglia phagocytosis as previously described 16,31. The background was subtracted from all fluorescent channels in Z-stacks of optical sections using ImageJ software. Subsequently, 3D volume surface renderings of each z-stack were created using Imaris software (Bitplane). Surface-rendered images were used to determine the volume of the microglia and lysosomes. The following equation was used to calculate the percent engulfment: volume of lysosomes (µm^3^) / volume of microglial cells (µm^3^).

### Reverse-transcription quantitative polymerase chain reaction (RT-qPCR)

The RT-qPCR analysis was performed as previously described 16. Briefly, total RNA was extracted from the retinas of rats using TRIzol. Total RNA (approximately 1-2 μg per 20 μl reaction) was reverse transcribed using a PrimeScript®RT Reagent Kit (TaKaRa Bio USA, Mountain View, CA, USA). SYBR Green qPCR Mix (Dongsheng Biotech, Guangdong, China) was used to perform quantitative PCR on a CFX96 Real-Time PCR System (Bio-Rad, Hercules, CA, USA) according to the manufacturer's instructions. The relative expression of the mRNAs was normalized to that of glyceraldehyde 3-phosphate dehydrogenase (GAPDH). All primers were purchased from Sangon Biotech (listed in Table S2).

### Flow cytometry

The flow cytometry analysis of retinas was performed as previously described 30. In brief, the retinas were dissociated into single-cell suspensions using the Neural Tissue Dissociation Kit - Postnatal Neurons (MACS; Miltenyi Biotec, cat. no. 130-094-802) according to its guidelines. The cell suspensions from retina or spleen tissues were filtered through a 70-mm strainer to prevent cell clumps. The cells were washed with PBS containing 1% FBS and stained with antibodies (Table S3) or isotype controls for 30 min at 4 °C. Flow cytometry data were acquired with a FACSCanto II (BD Biosciences) and analyzed with FlowJo software.

### Electroretinography (ERG)

ERG was performed using previously described methods 16. Briefly, gold wire loops were simultaneously used to record the corneal ERG responses from both eyes. Scotopic, rod-mediated responses were obtained from 12h dark-adapted rats at intensities of -4.5, -2.5, -0.5, -0.02, 0.5, and 1 log(cd·s·m^-2^). Photopic, cone-mediated responses were obtained from 0.5h light-adapted animals at intensities of 0.5 and 1 log(cd·s·m^-2^). The RETIscan system (Roland Consult, Brandenburg, Germany) was used to acquire the data, which were processed using Igor software.

### Optomotor response for visual acuity

The optokinetic head-tracking test was performed as previously described 32 and is illustrated in Figure 4J. Briefly, animals were dark-adapted for 12 h, and measurements for both eyes of each animal were recorded. A platform was set in the center of four inward-facing computer monitor screens with an infrared video camera overhead. The rats were placed on the platform and exposed to rotated grating stimuli (12 °/s) using a staircase paradigm program written by MATLAB showing spatial frequencies (0.05, 0.075, 0.1, 0.2, 0.3, 0.4, 0.5, and 0.6 cycles/degree) in 100% contrast to measure the visual acuity in both eyes. The head tracking response was driven by clockwise and counterclockwise rotations to evaluate each eye. The highest spatial frequency with a response was recorded as the visual acuity of each eye.

### RNA-Seq and bioinformatics analysis

For convenience, we labeled the control rat group as “Control P50”, the RCS rat group as “RCS P50” and the PLX3397-treated RCS rat group as “RCS+PLX P50”. Three biological replicates were used for the sample analysis in the “Control P50” and “RCS P50” groups, and two biological replicates were used for the sample analysis in the “RCS+PLX P50” group. RNA from retinal tissue samples at P50 was extracted using TRIzol^TM^ (Invitrogen, Carlsbad, CA, United States). mRNA was enriched with oligo (dT) magnetic beads and fragmented into short fragments using fragmentation buffer. The fragments were enriched by PCR amplification to construct transcriptome libraries. Primary raw reads produced by HiSeq 4000 (Illumina) were qualified and filtered to obtain clean reads. Gene expression levels were shown as the FPKM value. We detected differentially expressed genes (DEGs) with DEG-seq as requested and considered adjusted P-values ≤ 0.05 to be indicative of DEGs. Gene Ontology (GO) annotation analysis was performed for DEGs. The Kyoto Encyclopedia of Genes and Genomes (KEGG) database was used to perform pathway analysis of these DEGs.

### Statistical analysis

Data were analyzed using an independent two-sample t-test, one-way analysis of variance (ANOVA), or two-way ANOVA with Prism (GraphPad) or SPSS 17.0 statistical software (SPSS Inc., Chicago, IL, USA). The data are presented as the means ± the standard deviations (SDs). P-values less than 0.05 were considered statistically significant.

## Results

### Resident microglia are gradually activated to the DAM phenotype in the photoreceptor layer of RCS rats

**Figure 1:**
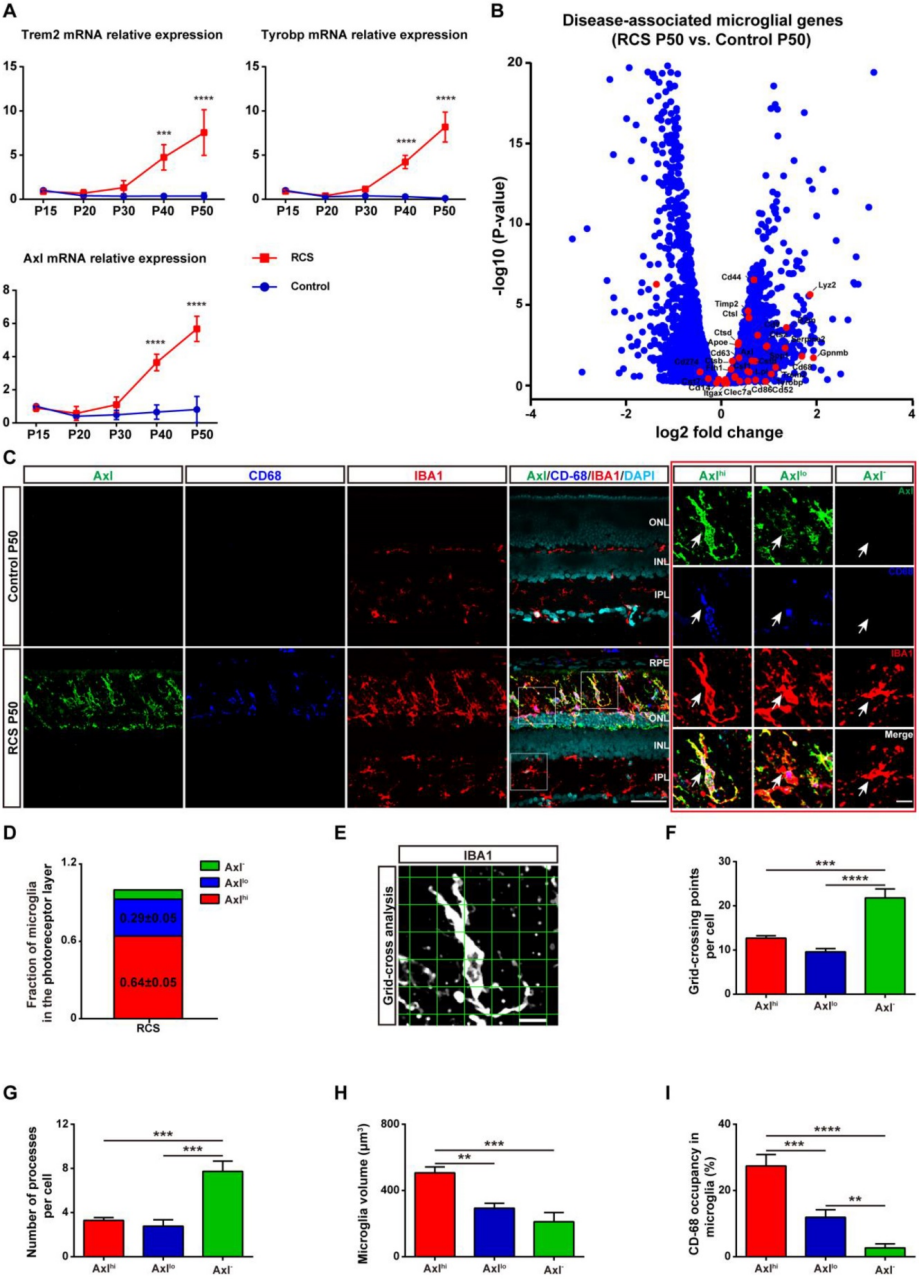
** DAM is activated in the photoreceptor layer of RCS rats.** (A) Relative gene transcript levels of Trem2, Tyrobp and Axl in the retinas of control rats and RCS rats are quantified by qPCR analysis. (N = 3 eyes from different rats, relative to control rats at P15). (B) Volcano plot showing the fold change in genes (x-axis, log2 scale) between RCS and control rat retinas and their significance (y-axis, -log10 scale). All genes are indicated by blue dots, and subsets of the disease-associated microglial genes are indicated by red dots. (C) Representative high-resolution confocal images showing immunofluorescent staining of Axl, CD68 and IBA1 in the retinas of control rats and RCS rats at P50. The right red panel shows the typical classification of microglial cells based on Axl expression in the retinas of RCS rats at a higher magnification. (D) Quantification of the proportion of Axl^hi^ (DAM), Axl^lo^ (intermediate) and Axl^-^ (homeostatic) microglia in the photoreceptor layer of RCS rats at P50 (N = 3 retinas from different rats, N = 8-12 images (213 × 213 µm) from each retina). (E) Representative images of microglia analyzed by grid (9 × 9 µm) crossing. (F) Statistical analysis of the grid-crossing points in each microglial cell in the retinas of RCS rats at P50 (N = 3 retinas from different rats, N = 25 (Axl^hi^), 15 (Axl^lo^) and 15 (Axl^-^) microglia from each retina). (G) Statistical analysis of the processes in each microglial cell in the retinas of RCS rats at P50 (N = 3 retinas from different rats, N = 25 (Axl^hi^), 15 (Axl^lo^) and 15 (Axl^-^) microglia from each retina). (H) Statistical analysis of the microglial volume in the retinas of RCS rats at P50 (N = 3 eyes from different rats, N = 11-15 (Axl^hi^), 11 (Axl^lo^) and 7 (Axl^-^) microglia from each retina). (I) Statistical analysis of CD-68 occupancy in the microglial volume in the retinas of RCS rats at P50 (N = 3 retinas from different rats, N = 11-15 (Axl^hi^), 11 (Axl^lo^) and 7 (Axl^-^) microglia from each retina). Abbreviations: RPE, retinal pigment epithelium; ONL, outer nuclear layer; OPL, outer plexiform layer; IPL, inner plexiform layer; INL, inner nuclear layer; GCL, ganglion cell layer. Scale bars, 50 µm (C), 10 µm (C) or 9 µm (E). Bars represent means; error bars represent SDs. *p < 0.05; **p < 0.01, ***p < 0.001, ****p < 0.0001 using one-way ANOVA (E-H) or two-way ANOVA (B).

As activated microglia were recently defined as DAM in a variety of neurodegenerative diseases, we determined whether microglia were similarly activated to the DAM phenotype during chronic photoreceptor degeneration. Trem2, Tyrobp, and Axl are identified as the key or marker genes in DAM activation 20,21; their mRNA levels were significantly increased in the retinas of RCS rats at P30, when obvious apoptotic rods were observed in the ONL 28. The increase in Trem2, Tyrobp, and Axl mRNA levels peaked at P50 (Figure 1A), by which point most photoreceptors were lost 28. By mRNA sequencing analysis (Figure S1), we found that many DAM genes in the RCS rat retinas were upregulated compared with those in the control group at P50 (Figure 1B), suggesting that the DAM signature was induced in the degenerating retina. Axl, a member of the TAM (Tyro3, Axl and Mertk) family, has previously been identified as a potential DAM marker for distinguishing subsets of microglia: Axl^hi^ (DAM), Axl^lo^ (intermediate stage), and Axl^-^ (homeostatic stage) 21,33. Retinal staining for IBA1, a classic microglial marker, and CD68, a marker of phagocytic activity, along with Axl, showed that the IBA1^+^ cells in the inner retinas of RCS rats and control rats were negative for Axl and CD68, suggesting a homeostatic state in these uninjured regions (Figure 1C). In contrast, IBA1^+^ cells double stained with Axl and CD68 were mainly located in the photoreceptor layers of RCS rats, particularly in the subretinal space (SRS) (Figure 1C). Moreover, many IBA1^+^ cells significantly expressed Trem2 in the degenerative region of RCS rats (Figure S2), suggesting that DAM activation was induced. At P50, 64% of microglia in the SRS and ONL showed the DAM phenotype, and 29% of microglia were at the intermediate stage (Figure 1D). Using standard morphological methods, we observed that the DAM and intermediate microglia were accompanied by classically activated microglia, which were characterized by a less ramified morphology, reduced branching sites, increased cell volume, and increased CD68^+^ lysosomal content (Figures 1E-I). Furthermore, some IBA1^+^ cells were only found to express low levels of Axl and CD68 (Figure S3), which suggests that microglia were activated to an intermediate state in the photoreceptor layers of RCS rats at P30. Our data indicated that the DAM phenotype was gradually activated during photoreceptor degeneration in RCS rats.

**Figure 2:**
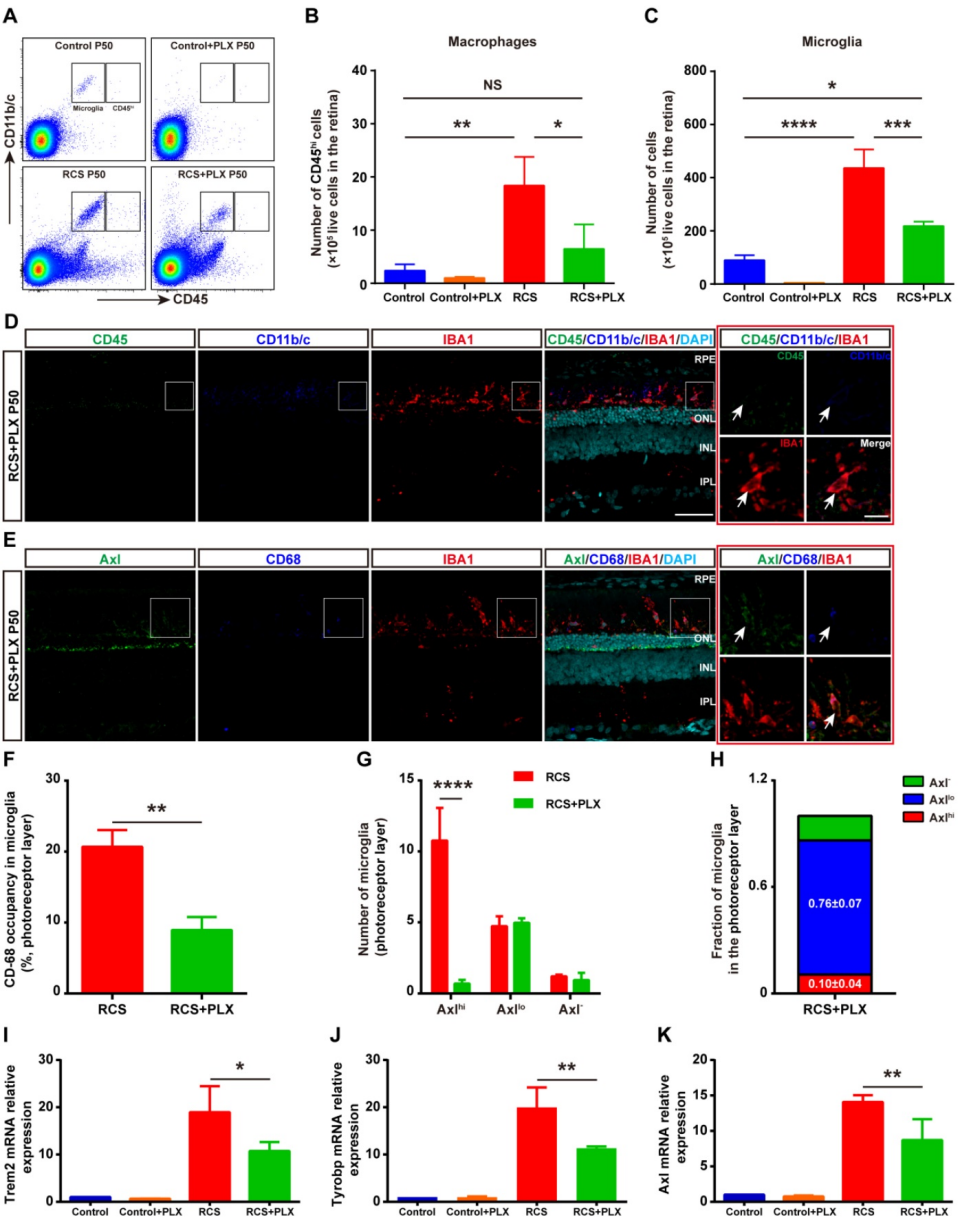
** The CSF1R inhibitor PLX3397 mainly depletes DAM in the photoreceptor layer of RCS rats.** (A) Representative flow cytometry plots showing the gating strategy of the microglia (CD11b/c^+^CD45^int^ and CD11b/c^+^CD45^lo^) and macrophage (CD11b/c^+^CD45^hi^) cell populations in the retinas of control rats, control rats treated with PLX3397, RCS rats and RCS rats treated with PLX3397 at P50. (B-C) Counts of microglia and macrophages in the retinas of control rats, control rats treated with PLX3397, RCS rats and RCS rats treated with PLX3397 at P50 (N = 3). The data represent the relative number of living cells (×10^5^) in the retina. (D) Representative high-resolution confocal images showing immunofluorescent staining of CD45, CD11b/c and IBA1 in the retinas of RCS rats treated with PLX3397 at P50. The right red panel shows the typical expression of CD45 and CD11b/c in the IBA1^+^ cells in the retinas of RCS rats treated with PLX3397 at a higher magnification. (E) Representative high-resolution confocal images showing immunofluorescent staining of Axl, CD68 and IBA1 in the retinas of RCS rats treated with PLX3397 at P50. The right red panel shows the typical expression of Axl and CD68 in the IBA1^+^ cells in the retinas of RCS rats treated with PLX3397 at a higher magnification. (F) Quantification of the volume (%) of microglia occupied by CD68-positive lysosomes in the outer retinas of RCS rats (N = 3 retinas from different rats, N = 18-22 microglia from each retina) and RCS rats treated with PLX3397 (N = 3 retinas from different rats, N = 14 microglia from each retina) at P50. (G and H) Quantification of the proportion and number of Axl^hi^ (DAM), Axl^lo^ (intermediate) and Axl^-^ (homeostatic) microglia in the outer retinas of RCS rats (N = 3 retinas from different rats, N = 8-12 images (213 × 213 µm) from each retina) and RCS rats treated with PLX3397 (N = 3 retinas from different rats, N = 8 images (213 × 213 µm) from each retina) at P50. (I-K) Trem2, Tyrobp and Axl gene relative transcript levels in the retinas of control rats, control rats treated with PLX3397, RCS rats and RCS rats treated with PLX3397 at P50 are quantified by qPCR analysis (N = 3 retinas from different rats, relative to control rats at P50). Abbreviations: SS, subretinal space; ONL, outer nuclear layer; OPL, outer plexiform layer; IPL, inner plexiform layer; INL, inner nuclear layer; GCL, ganglion cell layer. Scale bar, 50 µm (D and E) or 10 µm (D and E). Bars represent means; error bars represent SDs. *p < 0.05; **p < 0.01, ***p < 0.001, ****p < 0.0001 using an independent two-sample t test (F and G) or two-way ANOVA (B, C, I, J and K).

In certain disease states, blood-derived monocytes have been suggested to infiltrate the retina and differentiate into macrophages, which are difficult to distinguish from resident microglia with conventional markers such as IBA1, CD68, and CD11b/c 6,34,35. To determine whether monocyte-derived macrophages (mo-MFs) were involved in DAM activation, the relative expression of CD45 was used to distinguish these cell populations by immunohistochemistry and flow cytometry 18,36-38. A few macrophages (CD45^hi^) were found to infiltrate the degenerating retina, which was located close to retinal pigment epithelia (RPE) of RCS rats at P50 (Figure S4 and Figures 2A-B). Furthermore, most IBA1^+^ and CD11b/c^+^ cells with increased expression of CD45 (CD45^int^) were primarily restricted to the SRS in the retinas of RCS rats (Figure S4); these cells are previously considered DAM 39. In summary, the DAM phenotype was gradually activated in the photoreceptor layer of RCS rats, a process in which mo-MFs likely have little contribution.

### Most DAM are depleted by the prolonged treatment with a CSF1R inhibitor in the photoreceptor layer of RCS rats

To address the contribution of DAM activation to photoreceptor degeneration, we treated RCS rats with the CSF1R inhibitor PLX3397 from P15 to P50 (Figure S5A). PLX3397 efficiently depleted approximately 60% of IBA1^+^ cells in the retinas of RCS rats; remaining IBA1^+^ cells were mainly localized in the photoreceptor layers (SRS and ONL) of RCS rats (Figure S5B). In contrast, PLX3397 depleted nearly 100% of microglia in the retinas of control rats (Figure S6). Thus, PLX3397 efficiently but incompletely depleted microglia in the degenerating region, suggesting that the CSF1R inhibitor has differential depletion effects on the microglial subtypes.

To explore how CSF1R inhibition selectively depleted specific microglial subsets during DAM activation, we focused on the identity of the residual IBA1^+^ cells in the photoreceptor layer of RCS rats. First, we determined whether mo-MFs can fill the SRS and ONL in the absence of microglia. Few CD45^hi^ macrophages were detected in the retinas of RCS rats that received the 35-day PLX3397 treatment using immunohistochemistry and flow cytometry (Figures 2A-D); these data excluded the distribution of macrophages as residual IBA1^+^ cells. The fluorescence intensity of CD45 in the surviving IBA1^+^ cells was at a level in between CD45^int^ and CD45^lo^ in RCS rat retinas, and few CD45^int^ microglia were found in the retinas of PLX3397-treated RCS rats (Figures 2D). We detected low levels of Axl and CD68 fluorescence intensity in most IBA1^+^ cells in degenerating retinas treated with PLX3397 (Figure 2E), suggesting an intermediate stage (Axl^lo^) in the residual microglia. A similar reduction was observed when the CD68 volume-occupying surviving microglia in the outer retinas were compared between PLX3397-treated RCS rats and untreated RCS rats (Figure 2F). Most DAM (Axl^hi^) in the photoreceptor layer of RCS rats were lost after 35 days of treatment with PLX3397, but the number of intermediate microglia (Axl^lo^) was not significantly influenced by PLX3397 (Figure 2G-H). Moreover, Trem2, Tyrobp and Axl mRNA expression was significantly reduced in the retinas of PLX3397 treated RCS rats compared with that of untreated RCS rats (Figures 2I-K). Together, our data suggested that the DAM (Axl^hi^) were depleted by sustained CSF1R-inhibitor treatment and that the intermediate microglia (Axl^lo^) were more resistant to this treatment in the photoreceptor layers of RCS rats.

### Sustained treatment with a CSF1R inhibitor aggravates photoreceptor degeneration

**Figure 3:**
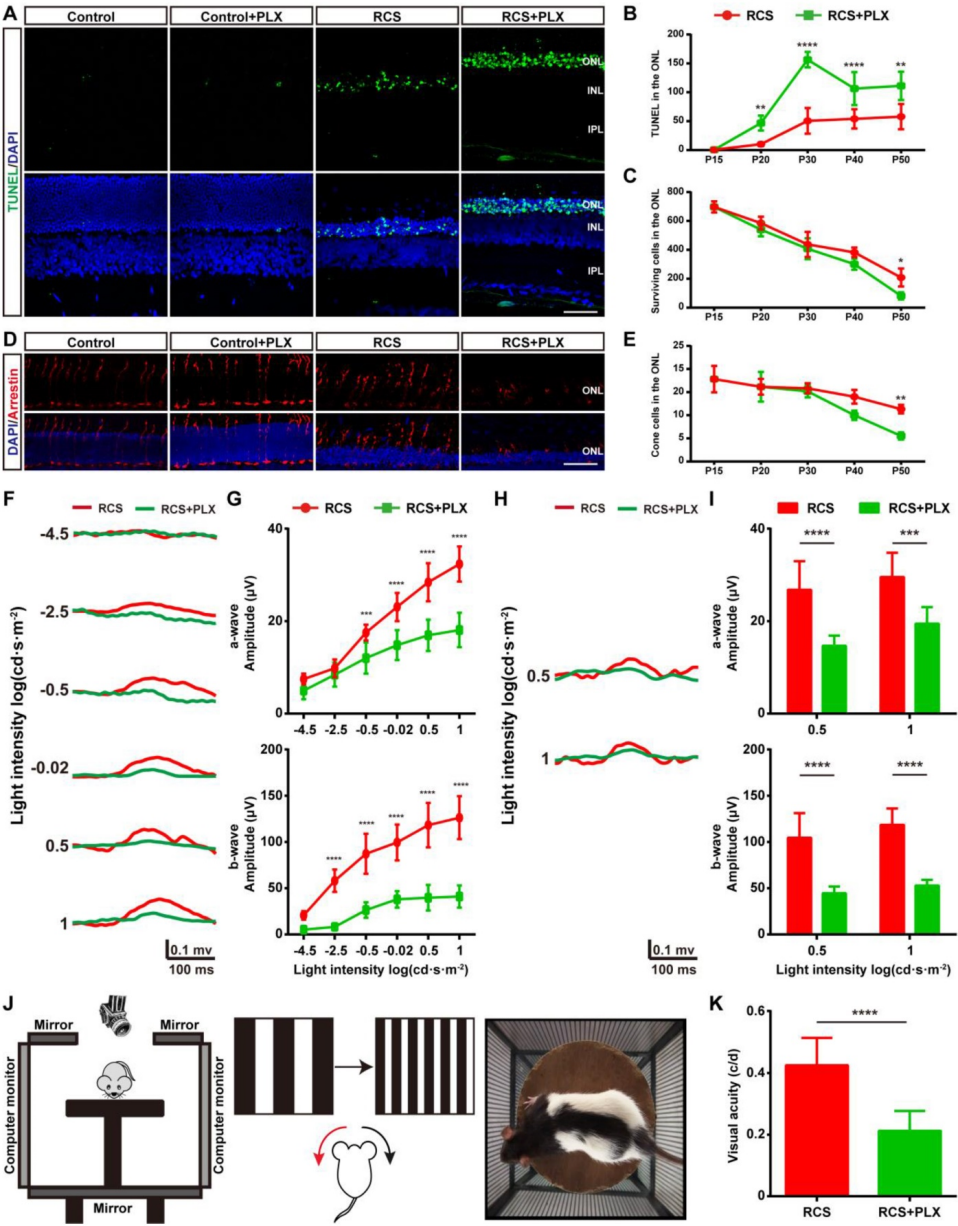
** Microglial depletion by PLX3397 treatment exacerbates photoreceptor degeneration and visual function deterioration.** (A) Representative high-resolution confocal images showing apoptotic photoreceptors using TUNEL (green) in the retinas of control rats, control rats treated with PLX3397, RCS rats and RCS rats treated with PLX3397 at P50. (B and C) Quantification of surviving and TUNEL-positive photoreceptor cells in the retinas of control rats, control rats treated with PLX3397, RCS rats and RCS rats treated with PLX3397 from P15 to P50 (N = 3 eyes from different rats, N = 5-10 images (213 × 213 µm) from each eye). (D) Representative high-resolution confocal images showed cones using arrestin staining in the retinas of control rats, control rats treated with PLX3397, RCS rats and RCS rats treated with PLX3397 at P50. (E) Quantification of cone cells in the retinas of control rats, control rats treated with PLX3397, RCS rats and RCS rats treated with PLX3397 from P15 to P50 (N = 3 eyes from different rats, N = 6-8 images (213 × 213 µm) from each eye) (F) Representative light-evoked scotopic ERG waveforms measured with six different light intensities (from -4.5 to 1 log(cd·s·m^-2^)) in RCS rats (red curve) and RCS rats treated with PLX3397 (green curve) at P50. (G) Average scotopic a-wave (top row) and b-wave (lower row) amplitudes elicited at six different light intensities (from -4.5 to 1 log(cd·s·m^-2^)) from RCS rats (N = 10) and RCS rats treated with PLX3397 (N = 8) at P50. (H) Representative light-evoked photopic ERG waveforms measured with 0.5 and 1 log(cd·s·m^-2^) in RCS (red curve) rats and RCS rats treated with PLX3397 (green curve) at P50. (I) Average photopic a-wave (top row) and b-wave (lower row) amplitudes elicited at 0.5 and 1 log(cd·s·m^-2^) from RCS rats (N = 6) and RCS rats treated with PLX3397 (N = 8) at P50. (J) Diagram showing the setup of the optokinetic response test. (K) Visual acuity analysis of the optokinetic response test from RCS rats (N = 8) and RCS rats treated with PLX3397 (N = 8) at P50. Abbreviations: ONL, outer nuclear layer; OPL, outer plexiform layer; IPL, inner plexiform layer; GCL, ganglion cell layer. Scale bar, 50 µm (G and J). Bars represent means; error bars represent SDs. *p < 0.05; **p < 0.01, ***p < 0.001, ****p < 0.0001 using an unpaired t test (F) or two-way ANOVA (B, D, H, I and K).

We then assessed the effect of PLX3397 treatment on photoreceptor degeneration in RCS rats to determine the contribution of DAM to this degeneration. We performed a TUNEL assay to examine whether DAM depletion affected photoreceptor death in RCS rats. When we quantified the number of apoptotic cells in the degenerating retina, significant increases were found in the number of apoptotic cells (TUNEL^+^) in the ONL of PLX3397-treated RCS rats from P20 to P50 (Figures 3A-B); however, an obvious decline in the number surviving photoreceptors was detected only after 35 days of PLX3397 treatment (Figure 3C). The cones degenerated in a manner resembling secondary injury in RCS rats after P40 (Figure S7). Similarly, a significant decrease in the quantity and disruption in the morphology of the surviving cones were found in the retinas of RCS rats treated with the CSF1R inhibitor at P50 (Figures 3D-E). Apoptotic photoreceptors and injured cones were not detected in the retinas of control rats treated with PLX3397 from P15 to P50 (Figures 3A and D), suggesting that microglia elimination by sustained treatment with PLX3397 accelerated rod and cone loss.

To verify whether the PLX3397 treatment impaired photoreceptor cell function and visual behavior in RCS rats, we performed functional studies that measured electroretinograms (ERGs) and optomotor responses at P50. Selective reductions in the scotopic a-wave responses were found at four high light intensities in the RCS rats treated for 35 days, along with a prominent decrease in scotopic ERG b-waves (Figures 3F-G). Similarly, a-wave and b-wave amplitudes in the photopic ERGs of treated RCS rats were decreased at P50 compared with those of the untreated groups, indicating a decline in cone function (Figures 3H-I). The optokinetic head-tracking response (OKR) test, an established simple and precise method for quantifying animal vision 40, was performed to explore the impact of DAM elimination on visual acuity in RCS rats (Figure 3J). The visual acuity of RCS rats given 35 days of PLX3397 treatment declined by approximately 50% at P50 compared to that of age-matched RCS rats without treatment (Figure 3K), showing that microglial depletion in the retinas of RCS rats by PLX3397 further enhanced the functional impairment of rods and cones.

**Figure 4:**
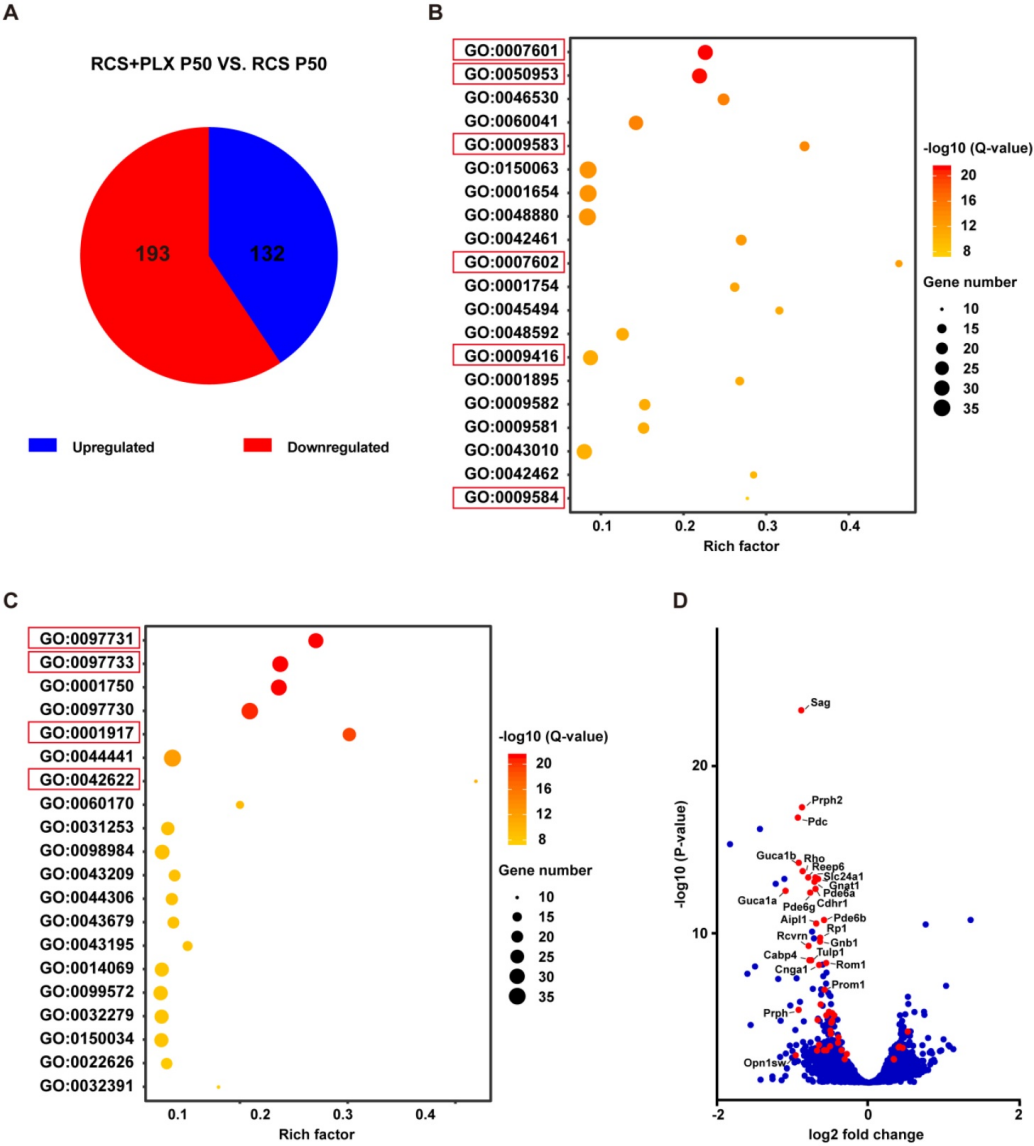
** Microglial depletion downregulates the expression of genes associated with photoreceptor function and components in the retinas of RCS rats.** (A) Pie chart of all DEGs (adjusted P-values ≤ 0.05). Red: gene downregulated by PLX3397; blue: gene upregulated by PLX3397 (N = 2 retinas from RCS rats treated with PLX3397, N = 3 retinas from RCS rats). (B) The 20 most significant biological processes from the GO enrichment analyses. Q-values represent the level of significance of enrichment. (C) The 20 most significant cellular components from the GO enrichment analyses. Q-values represent the level of significance of enrichment. (D) Volcano plot showing the fold change in genes (log2 scale) between the retinas of RCS rats treated with PLX3397 and RCS rat (x-axis) and their significance (y-axis, -log10 scale). All genes are indicated by blue dots, and DEGs associated with photoreceptor function and components enriched in the pathways underlined in panels B and C are indicated by red dots.

Next, we analyzed the effect of PLX3397 treatment on photoreceptor degeneration at the transcriptomic level to determine whether microglia elimination was also associated with a change in gene expression in photoreceptors. The gene expression profile of the retinas of PLX3397-treated RCS rats at P50 was compared with that of the retinas of untreated RCS rats. The mRNA sequencing results identified a total of 325 significantly DEGs (adjusted P values ≤ 0.05). Of those, 132 DEGs were expressed at significantly higher levels, and 193 DEGs were expressed at significantly lower levels in the PLX3397 group than in the untreated group (Figure 4A). Next, we conducted a GO enrichment analysis of biological processes (Figure 4B) and cellular components (Figure 4C), and focused on the 20 most significant pathways; we found that many highly significant pathways, such as pathways related to visual perception, sensory perception of light stimuli, photoreceptor outer segment, and photoreceptor cell cilium, were related to photoreceptor function and components. Importantly, most of the DEGs in these above pathways were downregulated following PLX3397 treatment (Figure 4D), indicating that sustained PLX3397 treatment impaired the gene expression of photoreceptor function and components in RCS rats at P50. Therefore, these results showed that CSF1R inhibition exacerbated photoreceptor degeneration, including secondary degeneration of cones in RCS rats. This finding indicated that certain microglial populations targeted by CSF1R inhibition, mainly DAM, attenuated disease progression in the photoreceptor layers of RP, implying a protective role of DAM in response to photoreceptor degeneration.

### DAM activation promotes microglia to clear dead cells and debris by increasing their phagocytic ability

Next, we examined the functional role of DAM activation in RP to gain insight into the mechanisms of photoreceptor protection. DAM are considered a disease-associated phagocytic cells that upregulate phagocytic, lysosomal, and endocytic genes conserved in neurodegenerative diseases 20,21. The microglia in the photoreceptor layer markedly upregulated the expression of Axl and CD68 during DAM activation (Figures 1A and C). When we compared the transcriptomes of retinas from RCS rats at P50 with those of the control rats, the lysosomal/phagocytic and endocytosis pathways were differentially regulated according to KEGG pathway analysis and GO enrichment analyses of biological processes and cellular components (Figure S1C). This finding indicated that activated microglia had a heightened ability for phagocytic clearance.

At P30, the reactive microglia migrated to the ONL and extended processes to touch apoptotic cell nuclei (Figure S8A). A significant fraction of microglia in the ONL contained lysosomes carrying cell nuclear fragments, as determined by the presence of DAPI^+^ nuclear material (Figures S8A-D), which is indicative of apoptotic cell clearance. During RP, detached debris from the photoreceptors accumulates in the SRS. Thus, the markers for rods (Gnat-1) and cones (arrestin) were used to detect the clearance of cell debris by microglia. At P30, several microglia moved into the SRS and projected their processes to internalize the outer segments of rods stained for Gnat-1 (Figures S8E, G, and H). Cones exhibited a normal morphology in the photoreceptor layer of RCS rats at P30. No interaction between IBA1 and arrestin was found (Figures S8F, I and J), indicating little direct interaction between stressed cones and microglia. This result suggested that the intermediate microglia mainly engulfed apoptotic rods at the early stage of photoreceptor degeneration.

**Figure 5:**
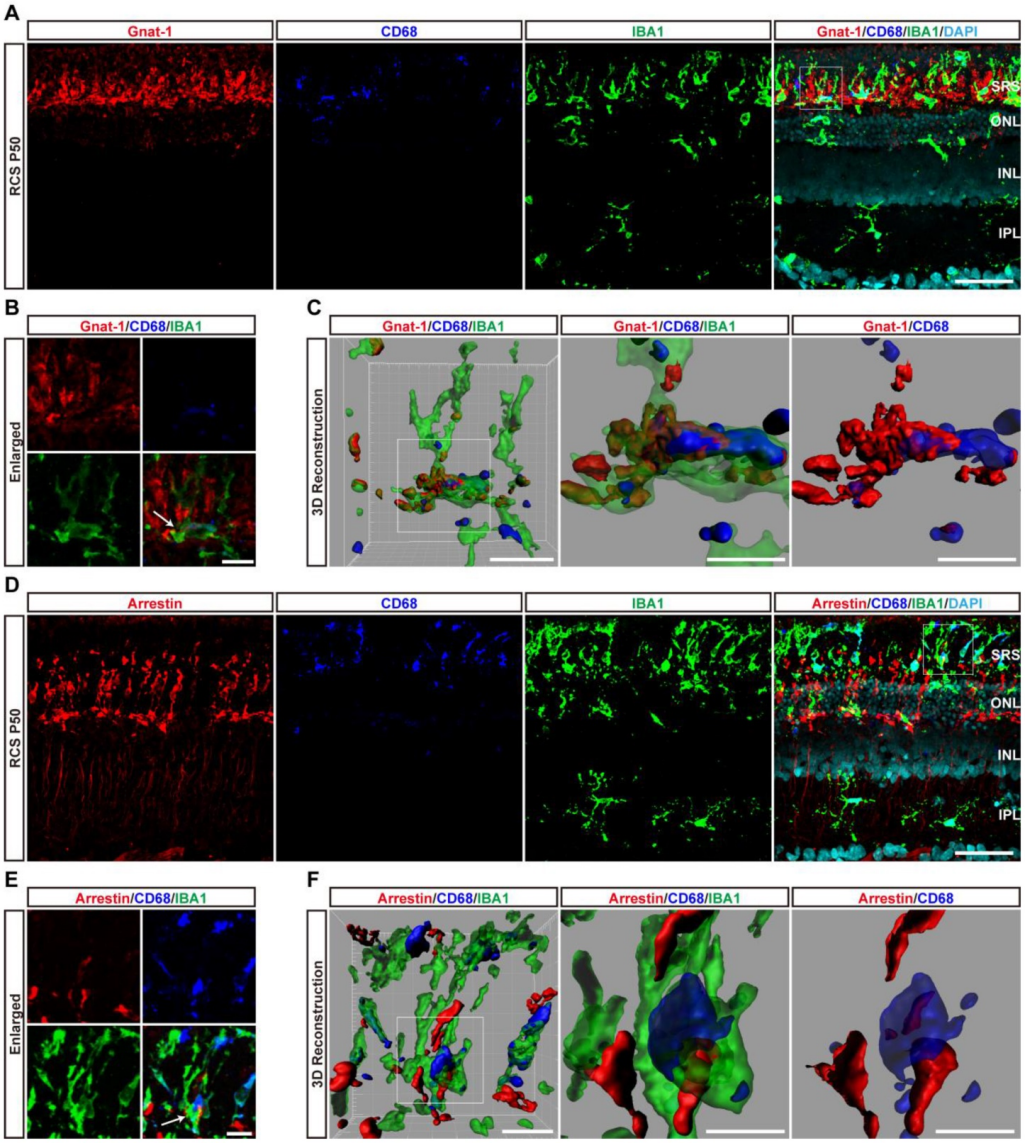
** DAM phagocytoses photoreceptor cell debris in the photoreceptor layer of RCS rats.** (A) Representative immunofluorescence images of Gnat-1 (red), CD68 (blue), IBA1 (green) and DAPI (cyan) in retinal sections from RCS rats at P50. (B) High-resolution confocal images from panel A are shown at a higher magnification. (C) Three-dimensional reconstruction and surface renderings of IBA1 (green), Gnat-1 (red) and CD68 (blue) in the retinas of RCS rats at P50. (D) Representative immunofluorescence images of arrestin (red), CD68 (blue), IBA1 (green) and DAPI (cyan) in retinal sections from RCS rats at P50. (E) High-resolution confocal images from panel D are shown at a higher magnification. (F) Three-dimensional reconstruction and surface renderings of IBA1 (green), arrestin (red) and CD68 (blue) in the retinas of RCS rats at P50. Abbreviations: SRS, subretinal space; ONL, outer nuclear layer; OPL, outer plexiform layer; IPL, inner plexiform layer; INL, inner nuclear layer. Scale bars, 50 µm (A and D) or 10 µm (B and E) or 5 µm (C and F).

At P50, DAM containing large CD68-positive lysosomes were mainly located in the SRS. Representative high-resolution confocal images and magnified 3D-reconstructed images clearly showed that many shedding outer segments from rods were contained in the processes and somas of activated microglia, with some even internalization by CD68-positive lysosomal compartments (Figures 5A-C). Similar engulfment was observed for cone debris by analyzing the colocalization of arrestin with IBA1 and CD68, which shed from degenerating cones (Figures 5D-F). Thus, these results suggested that DAM activation mainly contributed to clearing dead photoreceptors and debris via engulfment in the retinas of RCS rats.

### DAM activation inhibits the increase of neutrophils in degenerating retinas by engulfing and suppressing CXCL1 secretion by Müller cells

**Figure 6:**
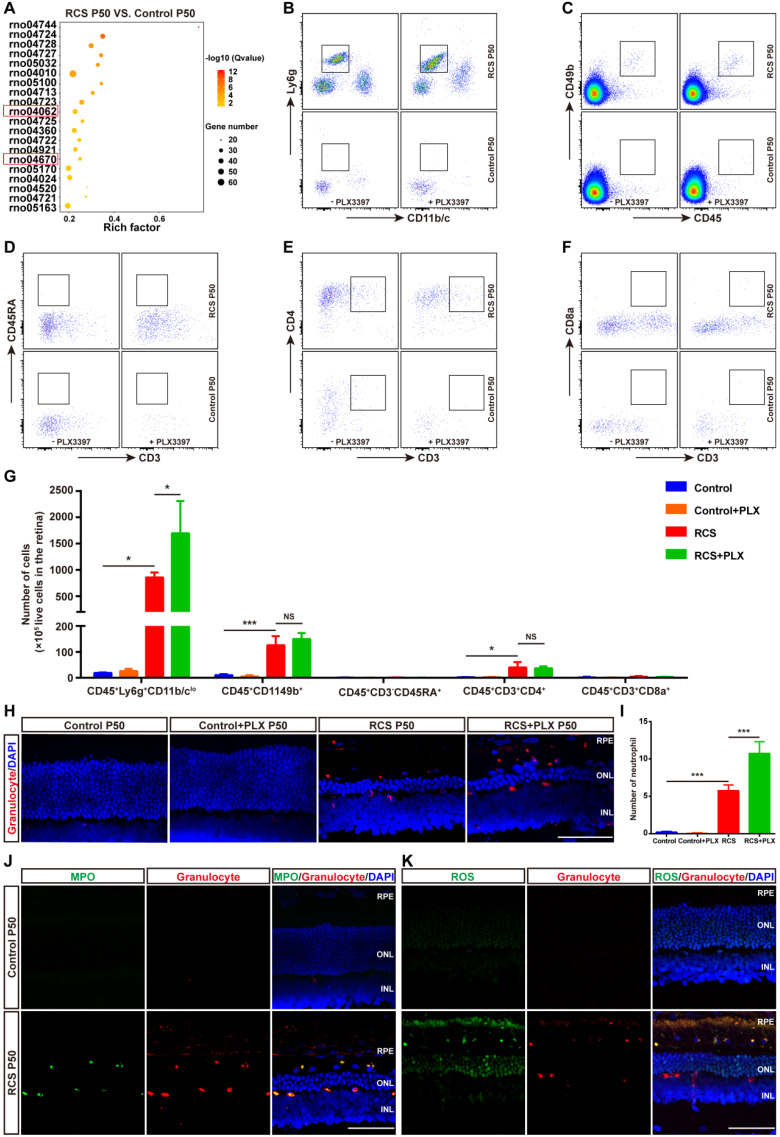
** CSF1R inhibitor-induced DAM depletion results in increased infiltrated neutrophils in the photoreceptor layer of RCS rats**. (A) KEGG pathway analysis of DEGs showing the top twenty most enriched clusters expressed in the retinas of control and RCS rats (N = 3 retinas from different rats). (B-F) Representative flow cytometry plots showing the gating strategy of leukocyte subpopulations isolated from retinas. Plots show the gating of neutrophils (CD45^+^Ly6g^+^CD11b/c^lo^), NK cells (CD45^+^ CD49b^+^), B cells (CD45^+^ CD45RA^+^CD3^-^), CD4^+^ T cells (CD45^+^ CD3^+^ CD4^+^) and CD8^+^ T cells (CD45^+^ CD3^+^ CD8^+^). (G) Quantification of retina-infiltrated leukocytes from control rats, control rats treated with PLX3397, RCS rats and RCS rats treated with PLX3397 at P50 (N = 3). The data represent the relative number of living cells (x 10^5^) per rat. (H) Representative high-resolution confocal images show neutrophils in the retinas of control rats, control rats treated with PLX3397, RCS rats and RCS rats treated with PLX3397 at P50. (I) Quantification of neutrophils in the retinas of control rats, control rats treated with PLX3397, RCS rats and RCS rats treated with PLX3397 at P50 (N = 3 eyes from different rats, N = 6-8 images (213 × 213 µm) from each eye). (J) Representative high-resolution confocal images show immunofluorescent staining of MPO and granulocytes in the retinas of control and RCS rats at P50. (K) Representative high-resolution confocal images show immunofluorescent staining of ROS and granulocytes in the retinas of control and RCS rats at P50. Abbreviations: SS, subretinal space; ONL, outer nuclear layer; OPL, outer plexiform layer; IPL, inner plexiform layer; INL, inner nuclear layer; GCL, ganglion cell layer. Scale bar, 50 µm (H, J and K). Bars represent means; error bars represent SDs. *p < 0.05; ***p < 0.001 using two-way ANOVA (G and I).

Another important function of DAM is the regulation of the immune response. We next explored the contribution of DAM activation to the immune microenvironment of degenerating retinas. By comparing the transcriptomes of retinas from RCS rats at P50 with control rats (Figure S1), the KEGG pathway analysis demonstrated that the chemokine signaling and leukocyte transendothelial migration pathways were among the top differentially regulated pathways (Figure 6A), suggesting an important role of leukocyte infiltration in disease progression. Therefore, we investigated whether DAM activation regulated the immune microenvironment by affecting leukocyte invasion. Flow cytometry analysis showed that photoreceptor degeneration induced a marked increase in the numbers of neutrophils (CD45^+^Ly6g^+^CD11b/c^lo^), NK cells (CD45^+^CD49b^+^), and CD4^+^ T cells (CD45^+^CD3^+^CD4^+^) in the retinas of RCS rats compared with those in control rats (Figures 6B, C, E, and G). B cells (CD45^+^CD45RA^+^CD3^-^) and CD8^+^ T cells (CD45^+^CD3^+^CD8^+^) were barely detected in the retinas of RCS and control rats (Figures 6D, F and G). Surprisingly, treating RCS rats with PLX3397 from P15 to P50 promoted a significant increase only in neutrophils (CD45^+^Ly6g^+^CD11b/c^lo^) in the retinas (Figures 6B and G). No changes in peripheral leucocytes were found in the retinas of control rats (Figures 6B-G), and no changes were observed in splenic macrophage and leukocyte populations after 35 days of PLX3397 treatment (Figure S9), implying that DAM activation mainly contributed to reduce infiltrated neutrophils in the injured retina.

To determine the distribution of neutrophils in the retinas of RCS rats, we performed immunohistochemistry using an anti-granulocyte antibody (HIS48), based on a previous study that reported that granulocyte (HIS48) is a useful marker for assessing neutrophils in rats 41. Granulocyte^+^ cells were found to be mainly located in the photoreceptor layers of RCS rats (Figure 6H and Figure S10). Treatment with PLX3397 from P15 to P50 significantly increased the number of granulocyte^+^ cells in the outer retinas of RCS rats (Figure 6I). Myeloperoxidase (MPO), another neutrophil-specific protein, is commonly used to quantify neutrophil infiltration in the CNS 42-44. By staining with MPO and granulocyte (HIS48), we found that granulocyte^+^ cells also expressed MPO and were distributed in the outer retinas of RCS rats (Figure 6J and Figure S10). In addition, these granulocyte^+^ cells produced many ROS (Figure 6K), revealing that infiltrated neutrophils can potentially damage photoreceptors during RP. Thus, DAM activation might play a key role in inhibiting the increase of retinal neutrophils, thereby suppressing damage to photoreceptor cells.

**Figure 7:**
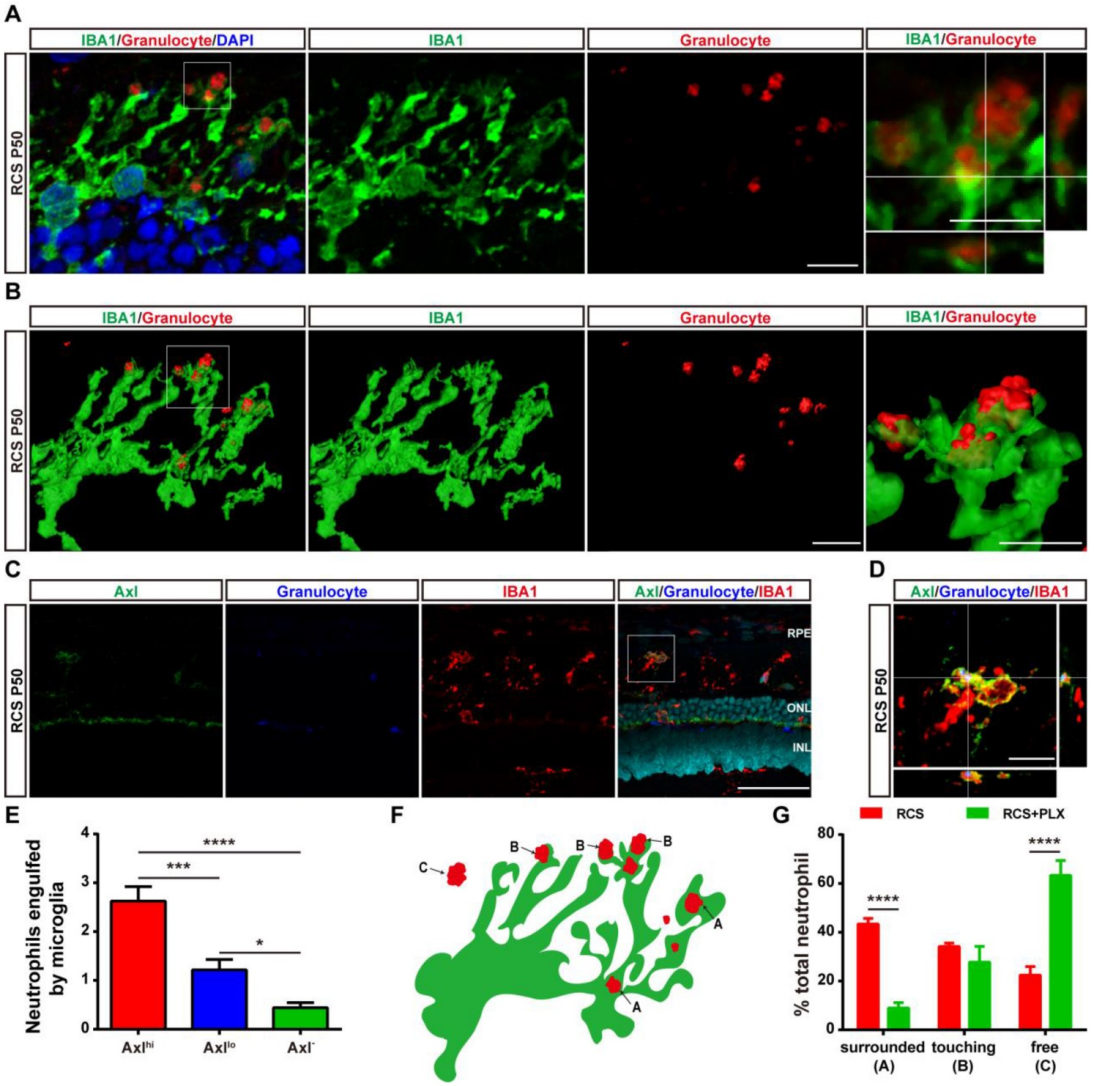
** DAM phagocytoses infiltrated neutrophils in the photoreceptor layer of RCS rats.** (A) Confocal images of microglial (green) cells engulfing neutrophils (red) in the retinas of RCS rats at P50. Reference boxes in the rightmost panels indicate orthogonal views of representative high-resolution confocal images at higher magnification. (B) Three-dimensional reconstruction and surface renderings of microglial cells (green) engulfing neutrophils (red) in the retinas of RCS rats at P50. (C) Representative high-resolution confocal images show immunofluorescent staining of Axl, granulocytes and IBA1 in the retinas of RCS rats at P50. (D) Orthogonal views of representative high-resolution confocal images show staining of Axl, granulocytes and IBA1 in the retinas of RCS rats at P50 at higher magnification. (E) Statistical analysis of neutrophils engulfed per microglia in the retinas of RCS rats at P50 (N = 3 eyes from different rats, N = 28-34 (Axl^hi^), 19-21 (Axl^lo^) and 13-15 (Axl^-^) microglia from each eye). (F) Schematic representation of the relationship between neutrophils and microglia illustrating: (A) a neutrophil completely phagocytosed into a microglial process (surrounded); (B) a microglial process engulfing a neutrophil (touching); and (C) no contact between a neutrophil and a microglial cell. (G) The proportion of each type of neutrophil shown in panel F in the outer retinas of P50 RCS rats (N = 3 eyes from different rats, N = 6-7 images (135 × 135 µm)) and treated rats (N = 3 eyes from different rats, N = 5 images (135 × 135 µm)). Abbreviations: RPE, retinal pigment epithelial; SS subretinal space; ONL, outer nuclear layer; OPL, outer plexiform layer; IPL, inner plexiform layer; INL, inner nuclear layer; GCL, ganglion cell layer. Scale bars, 50 µm (C) or 10 µm (A, B and D). Bars represent means; error bars represent SDs. *p < 0.05; ***p < 0.001; ****p < 0.0001 using one-way ANOVA (E) or two-way ANOVA (G).

As previous studies have shown that microglia can engulf infiltrated neutrophils in the injured brain 45, we investigated whether DAM decreased the number of infiltrated neutrophils by phagocytosis in the degenerating retina. Immunohistochemistry analysis showed that numerous microglial cell processes surrounded or contacted granulocyte^+^ cells (Figure 7A). 3D reconstructions of confocal images showed that microglia developed phagosomes by surrounding and contacting granulocyte^+^ cells (Figure 7B). To further determine whether the subtype affected microglia's neutrophil engulfment abilities, we observed relationships between microglial subpopulations and neutrophils by staining with Axl, IBA1, and HIS48. More neutrophils were engulfed by Axl^hi^ (DAM) microglia in the retinas of RCS rats (Figures 7C-E), suggesting that DAM activation enhanced the ability of microglia to clear infiltrated neutrophils. Treatment with the CSF1R inhibitor reduced the proportion of phagocytized neutrophils and increased the proportion of free neutrophils (Figures 7F-G), which indicated that suppression of DAM activation by the CSF1R inhibitor impaired the removal of infiltrated neutrophils. Thus, DAM activation decreased the number of infiltrated neutrophils by engulfing them in the photoreceptor layer of the degenerating retina.

**Figure 8:**
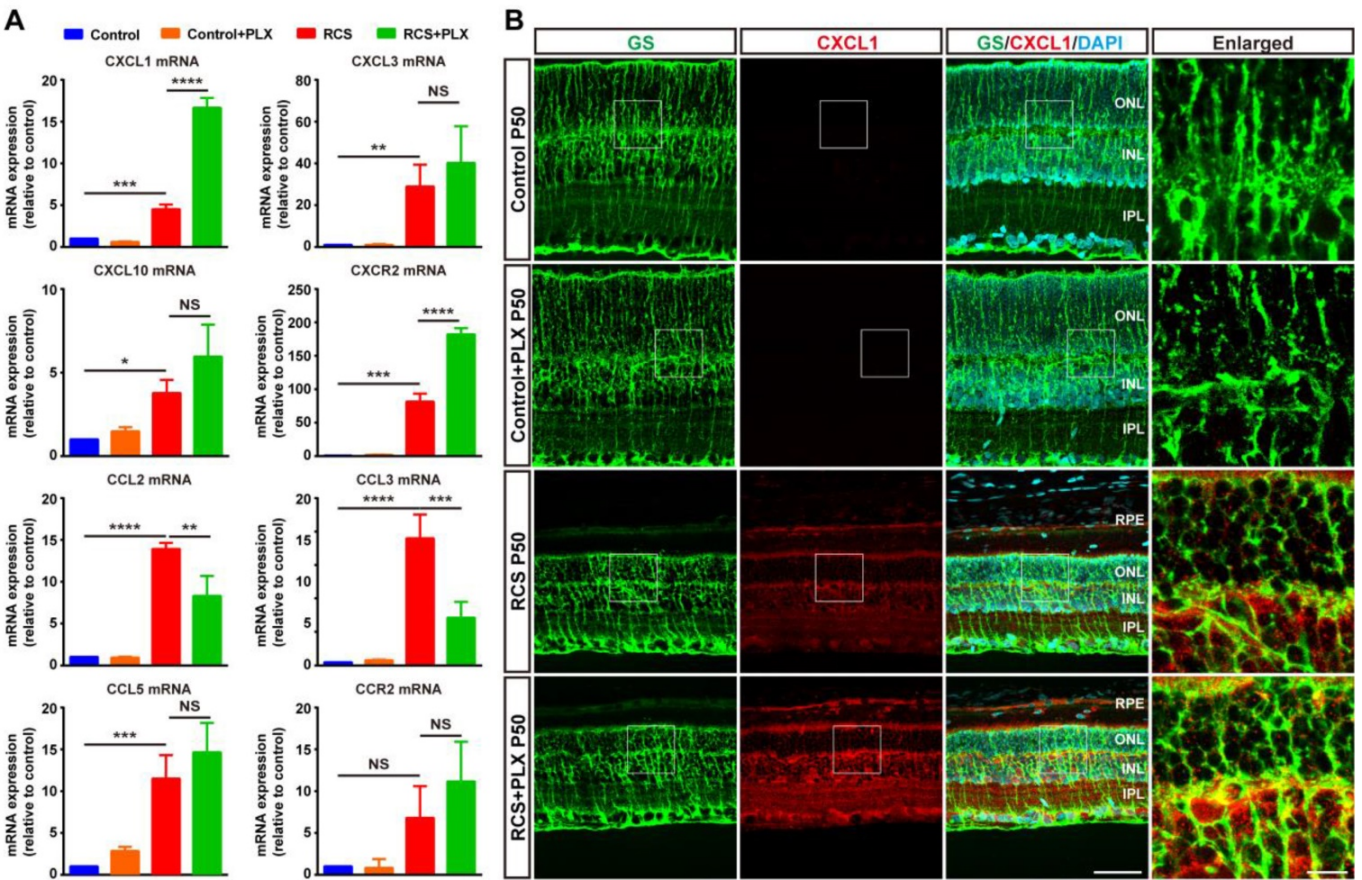
** Microglial elimination by PLX3397 promotes the secretion of the neutrophil chemoattractant CXCL1 by Müller cells.** (A) Chemokine and receptor gene relative transcript levels in the retinas of control rats, control rats treated with PLX3397, RCS rats and RCS rats treated with PLX3397 at P50 were quantified by qPCR analysis (N = 3 eyes from different rats, relative to control rats at P50). (B) Confocal images of GS (green) and CXCL1 (red) in the retinas of control rats, control rats treated with PLX3397, RCS rats and RCS rats treated with PLX3397 at P50. Reference boxes in the rightmost panels indicate orthogonal views of representative high-resolution confocal images at higher magnification. Abbreviations: RPE, retinal pigment epithelial; SS, subretinal space; ONL, outer nuclear layer; OPL, outer plexiform layer; IPL, inner plexiform layer; INL, inner nuclear layer; GCL, ganglion cell layer. Scale bar, 50 µm (B) or 10 µm (B). Bars represent means; error bars represent SDs. *p < 0.05; ***p < 0.001; ****p < 0.0001 using paired-samples t test or two-way ANOVA (A).

The selective migration of neutrophils depends on specific molecules and chemokines emerging after photoreceptor degeneration 46. To assess whether microglial activation affected the process of neutrophil recruitment to the retina from peripheral circulation, we examined the effect of PLX3397 treatment on neutrophil-attracting chemokines in the retinas of RCS rats. Although the secretion of many chemokines and cytokines was increased following retinal damage, treatment with PLX3397 dramatically increased the mRNA levels of C-X-C motif ligand 1 (CXCL1), a chemokine that mainly recruits neutrophils, and its receptor CXCR2 in the RCS rat retinas (Figure 8A). Staining for CXCL1 and glutamine synthetase (GS) in rat retinas revealed that Müller cells were a major source of retinal CXCL1 during RP and that microglial elimination increased the expression of CXCL1 by Müller cells (Figure 8B). In summary, these results indicated that DAM activation inhibited the increase of infiltrated neutrophils, harmful leukocytes that produce factors that damage photoreceptors in the retinas of RCS rats.

## Discussion

**Figure 9:**
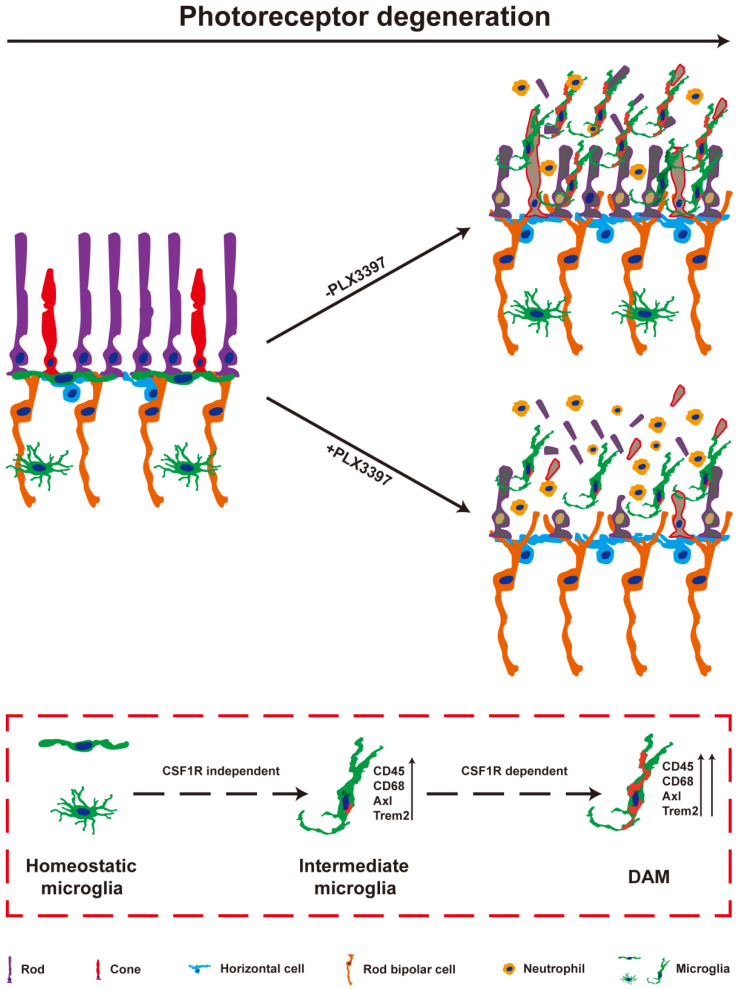
** DAM is activated in the photoreceptor layer of RCS rats in a CSF1R-dependent pathway and suppresses photoreceptor degeneration by removing dead photoreceptors and infiltrated neutrophils.** In a variety of neurodegenerative diseases, activated microglia have been identified as DAM, showing a conserved transcriptional signature. During RP, apoptotic photoreceptors also promote DAM activation, switching from a homeostatic stage to an intermediate phenotype and then to the DAM phenotype. Initial activation following signals associated with RP pathology induced microglia into an intermediate phenotype, which is independent of CSF1R signaling for activation, and survival. However, the transition from this intermediate phenotype to the fully activated DAM phenotype may be a CSF1R-dependent transition involving the upregulation of the phagocytic ability to clear dead photoreceptors and infiltrated neutrophils. Treatment with a CSF1R inhibitor mainly eliminated DAM in the photoreceptor layer of RCS rats, which aggravated the degeneration of photoreceptors, suggesting that DAM play a protective role during RD.

In the present study, we observed the pathways of microglial activation and the roles of activated microglia in the process of photoreceptor degeneration in an RP model. The microglia in the photoreceptor layer of RCS rats were gradually activated to the intermediate stage and then to DAM as photoreceptor death increased. Continuous treatment with a CSF1R inhibitor during DAM activation depleted most DAM. Importantly, sustained treatment with the CSF1R inhibitor exacerbated the loss and dysfunction of photoreceptors, implying that DAM might protect against photoreceptor degeneration. Furthermore, DAM were found to promote the clearance of dead cells and inhibit the increase of infiltrated neutrophils, which might be the important mechanism of delaying photoreceptor loss by decreasing secondary damage (Figure 9). Thus, microglia were gradually activated to DAM in the degenerating retina to support photoreceptor survival.

DAM activation is a conserved event in neurodegenerative conditions. Here, we further confirmed that DAM were activated in the photoreceptor layers of RCS rats. Subretinal microglia in the light damage model of acute photoreceptor degeneration and the Rho^P23H/WT^ model of retinitis pigmentosa have been shown to have overlapping expression signatures with DAM, including upregulation of Cd68, Axl, Tyrob, Apoe, Spp1 and Trem2 and downregulation of Tmem119, P2ry12, Cx3cr1, and Siglech 5. A similar change in molecular signatures of disease model-associated microglial clusters appears during choroidal neovascularization 47. We also found upregulation of the DAM gene signature in RCS rats, suggesting that DAM activation is a common pathological change during photorecetpor degeneration. In the present study, microglia migrated into the photoreceptor layer and were activated to an intermediate stage from P15 to P30, a time at which a few degenerating rods started to undergo apoptosis. When photoreceptor apoptosis reached its peak and many apoptotic photoreceptors were found in the photoreceptor layer (from P30 to P50), most microglia were further activated to DAM. Our results imply an apparent correlation between DAM activation and photoreceptor apoptosis. Furthermore, developmental apoptosis in the early postnatal retina is necessary for the DAM-related signature in retinal microglia 26, suggesting that apoptotic photoreceptors may be a major driver of the disease-related profile during RP. In the light damage model and the Rho^P23H/WT^ model of retinitis pigmentosa, microglia migrated to the SRS and adhered to the apical RPE, while a few mo-MFs occupied the neuroretina and neither migrated into the SRS nor adhered to the RPE 5. In AMD models, lesions affecting the overlaid RPE and photoreceptors, microglia constitute a major cell population in the diseased retina and RPE, along with a few recruited monocytes 47,48. A few CD45^hi^ cells were found in the retinas of RCS rats, implying that DAM might be derived from resident microglia. However, determining the isolated role of microglia or mo-MFs in the brain and/or retina has proven to be technically challenging due to lack of univocal markers. Mo-MFs have been suggested to overlap phenotypically with microglia and the CD45 relative expression level is not an accurate measurement. Thus, new technologies or cell markers are expected to distinguish activated microglia from mo-MFs in future studies.

CSF1R, a receptor tyrosine kinase, is important for microglial development, survival, and distribution 49,50. Inhibition of CSF1R signaling leads to complete depletion of microglia in the normal retina 51. However, we observed that a cluster of microglia persisted after sustained inhibition of CSF1R in the ONL and SRS of RCS rats. We determined that mo-MFs could not fill the photoreceptor layers after microglial depletion, a similar conclusion to those reached in other models of photoreceptor degeneration 5. We identified these residual microglia in an intermediate state, suggesting that intermediate microglia are more resistant to PLX3397 treatment during DAM activation 52. Concurrently, we observed near-complete depletion of DAM in the retinas of RCS rats treated with PLX3397 from P15 (a time before microglia were activated) to P50 (a time when many microglia were activated to the DAM phenotype). When microglia have been activated to the DAM phenotype, they do not require CSF1R signaling for survival 26,52. Thus, it is intriguing to speculate that DAM ablation by CSF1R inhibition might be due to interruption of the transition from the intermediate phenotype to the DAM phenotype. CSF1R has been shown to have prodifferentiative, pro-proliferative, and prosurvival functions by regulating tyrosine phosphorylation, activation, and the expression of multiple proteins 49,53-56. Importantly, CSF1R overlaps and interacts with TREM2 signaling 54,57, a receptor required to sustain the transition from the intermediate phenotype to the DAM phenotype 21. In the EAE model, CSF1R stimulation leads to expansion of the CD11c^+^ microglial subpopulation 58, which has been identified as a DAM population 20; this suggests that CSF1R signaling may be directly linked to DAM activation. Thus, we hypothesized that CSF1R signaling is necessary for the transition from the intermediate phenotype to the fully activated DAM phenotype and that it plays a key role, similar to that of TREM2. Our results and those of other studies demonstrate how CSF1R inhibition depletes certain microglial subsets, while selectively sparing other subsets, depending on the treatment time and the disease stage.

Taken together with our previous research, our results indicate that microglial elimination by the CSF1R inhibitor before P40 did not significantly affect the survival and function of degenerative photoreceptors in RCS rats 16, suggesting that early intermediate microglial activation might have little contribution to photoreceptor degeneration, particularly rod degeneration caused by primary defects. However, treatment with the CSF1R inhibitor from P15 to P50 significantly reduced the number of surviving photoreceptors (rods and cones) and impaired the function and genetic expression of photoreceptors throughout the retinas of RCS rats. Our previous study found that PLX3397 per se had no effect on photoreceptor survival and function in control rats 16, suggesting that DAM depletion by the CSF1R inhibitor might exacerbate photoreceptor degeneration. Consistent with this theory, conditional depletion of microglia prior to exposure to light damage (LD) or in P23H mice indicates that subretinal microglia, whose transcriptional profile exhibits similarities to that of DAM, help maintain the function and survival of photoreceptors by preserving RPE 5. These results suggest that DAM play a protective role in restricting photoreceptor degeneration. In previous studies, several inhibitors have been used to suppress the activation of microglia and delay the photoreceptor degeneration. For example, dexamethasone is found to decrease retinal inflammation when it is applied at the peak of retinal degeneration in Rd10 mice and protects the cones and rescues visual function 59. A second-generation tetracycline, minocycline, is reported to inhibite the activation of microglia through anti-inflammatory mechanisms, thus protecting photoreceptors against subretinal hemorrhage 60, diabetic macular edema 61 and RP 11. Dexamethasone and minocycline partially inhibit the function of microglia; recently, the well-characterized CSF1R inhibitors PLX3397 and PLX5622 are found to induce robust depletion of microglia. These varied results of different microglial inhibitors suggest that microglial responses to pathology might be complex and characterized by several disease-associated clusters of microglia.

We then discovered how DAM activation protected the photoreceptor. The rapid accumulation of dead photoreceptors and debris in the absence of microglia suggested one possible clue: the clearance of dead photoreceptors and debris. Microglia play a role in debris clearance, and their protective role has been reported in other models of photoreceptor degeneration, including LD, retinal detachment and retinitis pigmentosa. Damage-associated molecular patterns (DAMPs) released by dead cells and debris stimulate proinflammatory cytokine activity or recruit immune cells, such as neutrophils and T cells, which act as endogenous danger signals and incite inflammation 62,63. Hence, we surmised that DAM activation increased microglia-dominant clearance of neuronal debris by upregulating genes related to the lysosomal/phagocytic pathways and endocytosis as a main mechanism of suppressing secondary damage to the retina. Moreover, phagocytosis of apoptotic cells strongly promotes DAM activation 19,20, which obviously increases microglial engulfment, thus forming a positive feedback loop. Little direct contact between microglia and stressed cones was found at the early stage (at P30) of RP, providing new evidence that microglial primary phagocytosis of nonapoptotic cells is specifically conferred to living stressed rods rather than cones, as only a minority of cones show “eat-me” signals 10. As members of the TAM (Tyro3, Axl and Mertk) family, Mer is expressed on microglia during homeostasis, and Axl expression is upregulated in DAM 20,21. The microglia in the retinas of RCS rats also carry the MERTK mutation, while Mertk-knockout microglia can be also activated to the DAM phenotype with a indeterminately attenuated response of DAM genes 33,64. Thus, the engulfment of dead cells and debris by microglia with high Axl expression in the retinas of RCS rats suggests that Axl might play a dominant role in DAM activation, while Mertk did little influence on this process.

The normal CNS, including the retina, is a region that typically excludes leukocytes, such as granulocytes, myeloid-derived cells, and lymphocytes, a process referred to as 'immune privilege' 65,66. However, peripheral immune cells have been reported to infiltrate the degenerating regions of retinas and brains 13,27. In the present study, we observed neutrophils, NK cells, and CD4^+^ T cells in the retinas of RCS rats, suggesting that peripheral immune cells are involved in RP. Notably, DAM activation was observed to inhibit the increase of infiltrated neutrophils in the degenerating retina by engulfing neutrophils and suppressing CXCL1 secretion by Müller cells. Likewise, similar microglia regulation of brain neutrophil accumulation is found in models of ischemic stroke 45,67. After acute brain damage, neutrophils are one of the first cell types that contribute to blood-brain barrier disruption, edema, and neuronal death by producing ROS, cytokines, and metalloproteases, and blocking neutrophils results in marked protection 45,68,69. Similarly, ROS and MPO were generated by the infiltrated neutrophils in the photoreceptor layers of RCS rats, suggesting that neutrophils caused secondary damage to surviving photoreceptors. Thus, the suppression of neutrophil infiltration may be another mechanism by which DAM activation inhibits the secondary degeneration of photoreceptors, especially cones. However, the inflammatory response of microglia and/or Müller cells and their interaction are extremely complicated and further study is needed to determine their contribution to photoreceptors in different models.

In summary, our study revealed the pathways of microglial activation and its contribution to the process of photoreceptor degeneration in RCS rats. Previous studies have suggested that DAM have the potential to restrict neurodegeneration, according to analysis of its transcriptome 70. We further provided evidence to support the neuroprotective effects of DAM activation in RP models and explored its potential mechanisms. Based on our findings, the regulation of DAM activation might provide a therapeutic approach for novel treatment strategies that aim to suppress secondary damage to surviving photoreceptors.

## Supplementary Material

Supplementary figures and tables.
