# Supplementary material for: Disease-associated microglial activation prevents photoreceptor degeneration by suppressing the accumulation of cell debris and neutrophils in degenerating rat retinas
Source: Theranostics. 2022 Mar 6;12(6):2687–706. doi: 10.7150/thno.67954 (PMC8965480; doi:10.7150/thno.67954)
Supplement: Supplementary file 1 — Supplementary figures and tables. [file thnov12p2687s1.pdf]

Supplementary materials

Tables

Table S1. Antibodies used for immunofluorescence staining.

Antibody	Company	Titer	Species	Cat#
IBA1	Wako Chemicals	1:500	Rabbit	019-19741
IBA1	Novus	1:200	Goat	NB100-1028
CD-68	Bio-RDA	1:500	Mouse	MCA341R
Arrestin	Millipore	1:500	Rabbit	AB15282
Axl	R&D Systems	1:50	Goat	AF854
CD11b/c	Millipore	1:500	Mouse	CBL1512
GS	BD Biosciences	1:500	Mouse	610517
CXCL1	Abcam	1:100	Rabbit	ab86436
Granulocyte	Novus	1:100	Mouse	NB100-64933
Myeloperoxidase	Abcam	1:100	Rabbit	ab9535
Gant-1	Santa Cruz	1:200	Rabbit	sc-389
CD45	Biolegend	1:50	Mouse	202205
Trem2	Abcam	1:50	Mouse	ab201621

Table S2. Primers used for RT-qPCR.

Name	Sequence
GAPDH-F	GCCCATCACCATCTTCCAGGAG
GAPDH-R	GAAGGGGCGGAGATGATGAC
IBA1-F	GGATTTGCAGGGAGGAAAAG
IBA1-R	TGGGATCATCGAGGAATTG
AXL-F	CAGGTACCGTGCCCGAAA
AXL-R	TCGCTGATGCCCAGACTGT
CSF1R-F	GCTCTGATGTCCTGCTCTGTGA
CSF1R-R	CCTGCACTCCATCCATGTCA
TREM2-F	AAGATGCTGGAGACCTCTGG
TREM2-R	GGATGCTGGCTGTAAGAAGC
TYROB-F	AGCCCTCCTGGTGCTTTCT
TYROB-R	GGCCTGTACGGGACTTAATC
CXCL1-F	GGCAGGGATTCACTTCAAGA
CXCL1-R	GCCATCGGTGCAATCTATCT
CXCL3-F	TCACTTCCATTCTGTTGCAG
CXCL3-R	CCTCCCTGTGACACTGAAGA
CXCL10-F	ATGAACCCAAGTGCTGCCGTC
CXCL10-R	TTAAGGAGCCCTTTTAGACCTTT
CXCR2-F	CCAAGCTGATCAAGGAGACC
CXCR2-R	GGGGTTAAGACAGCTGTGGA
CCL2-F	ATGCAGTTAATGCCCCACTC
CCL2-R	TTCCTTATTGGGGTCAGCAC

CCL5-F	GAGTAGGGGGTTGCTCAGTG
CCL5-R	GCCAACCCAGAGAAGAAGTG
CCR2-F	CCATTCTGGGCTCACTATGC
CCR2-R	AGGGCCACAAGTATGCTG

Table S3. Antibodies used for flow cytometry.

Antibody	Company	Cat#
PE anti-rat CD45	Biolegend	202207
Alexa Fluor® 647 anti-rat CD11b/c	Biolegend	201814
FITC Hamster Anti-Rat CD49b	BD Biosciences	561891
APC anti-rat CD45RA	Biolegend	202313
Anti-Ly6g [RB6-8C5] (FITC)	Abcam	ab25024
Alexa Fluor® 488 anti-rat CD3	Biolegend	201406
APC anti-rat CD4 Antibody	Biolegend	201509
APC anti-rat CD8a Antibody	Biolegend	200609

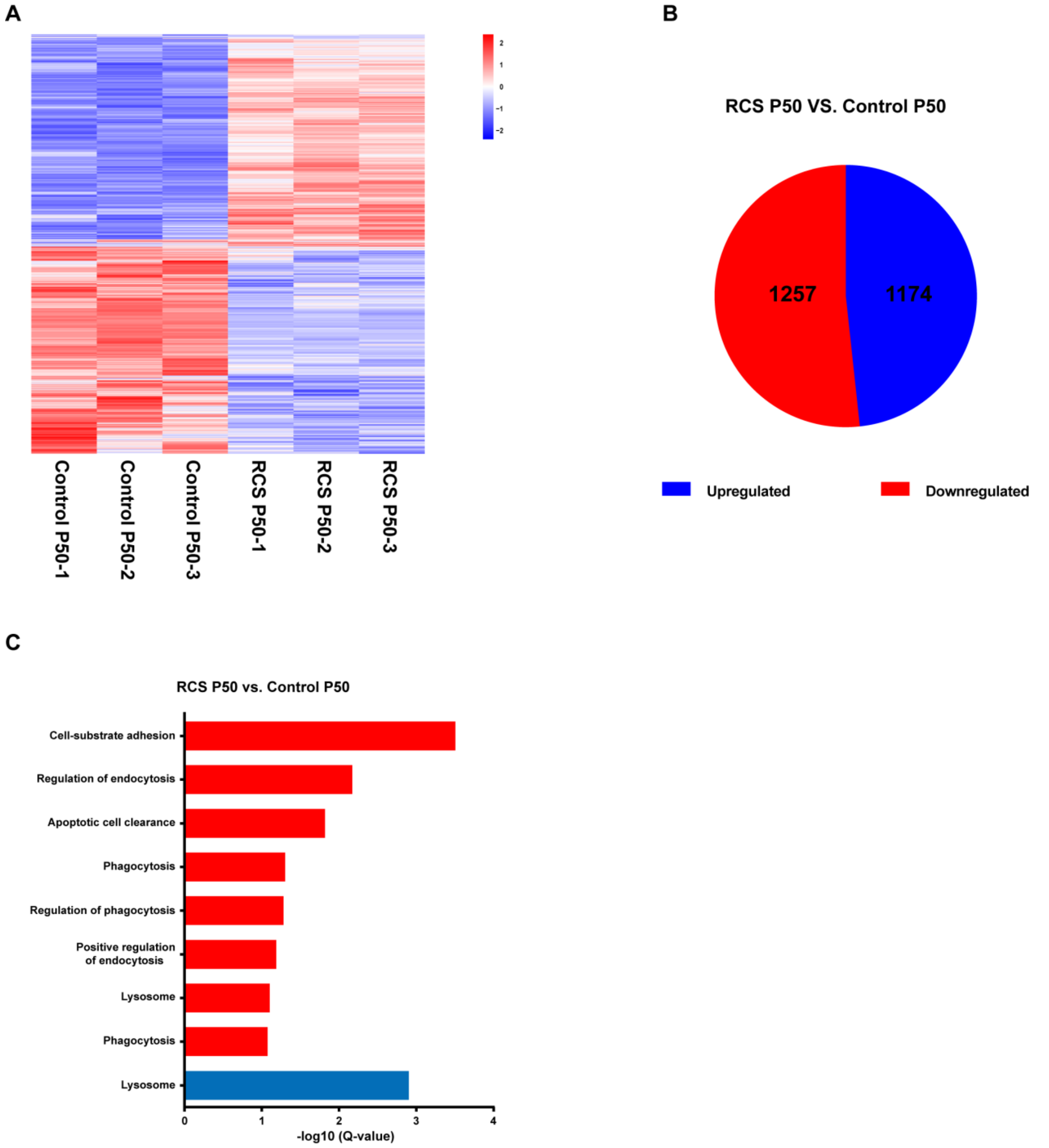

Supplementary Figure 1. RNA-Seq results of retinas from the control rats and RCS rats

(A) Hierarchical clustering of DEGs in the control and RCS groups.

(B) Pie chart of all DEGs (adjusted P-values ≤ 0.05). Red: gene downregulated by RD; blue: gene upregulated by RD (N = 3 retinas from RCS rats, N = 3 retinas from control rats).

(C) The lysosomal/phagocytic pathways and their related processes in the GO enrichment analyses (red bar) and KEGG enrichment analyses (blue bar) differentially enriched in RCS and control rats. Q-values represent the level of significance of enrichment.

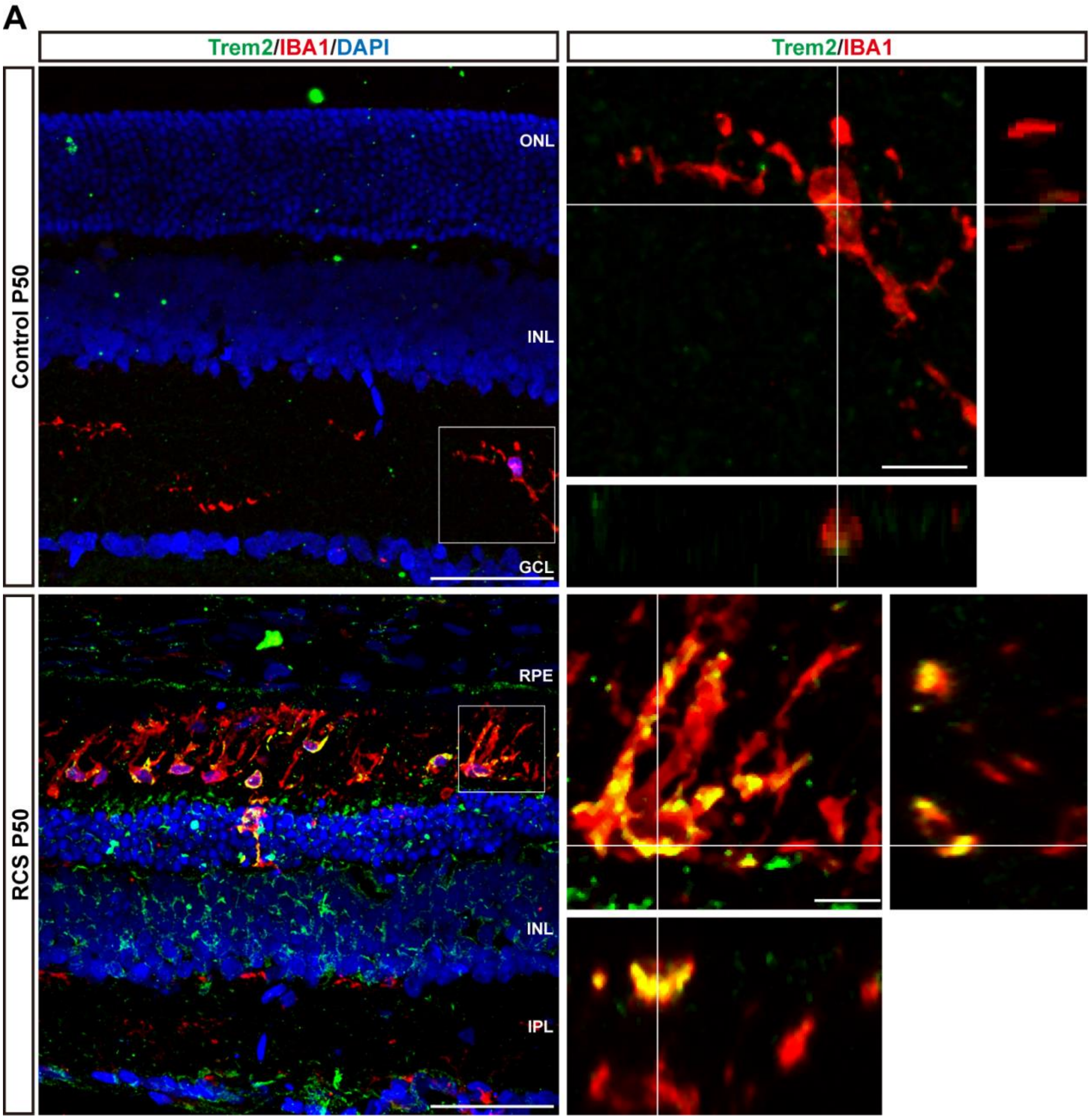

Supplementary Figure 2. Microglia are activated to the DAM phenotype in the outer retina of RCS rats at P50.

(A) Representative high-resolution confocal images showing immunofluorescent staining of Trem2 and IBA1 in the retinas of control rats and RCS rats at P50. The right panel shows orthogonal views of high-resolution confocal images in the left inset boxes. Scale bars, 50 µm or 10 µm.

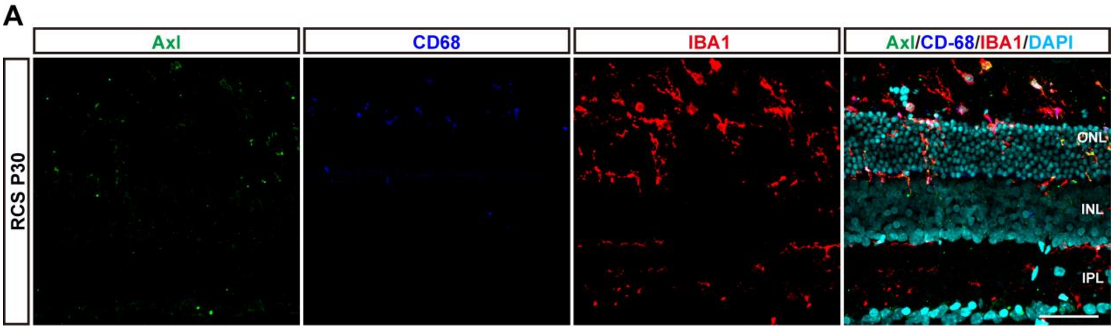

Supplementary Figure 3. Microglia are mainly activated to the intermediate phenotype in the outer retinas of RCS rats at P30.

(A) Representative high-resolution confocal images showing immunofluorescent staining of Axl, CD68 and IBA1 in the retinas of RCS rats and RCS rats treated with PLX3397 at P30. Scale bar, 50 μm.

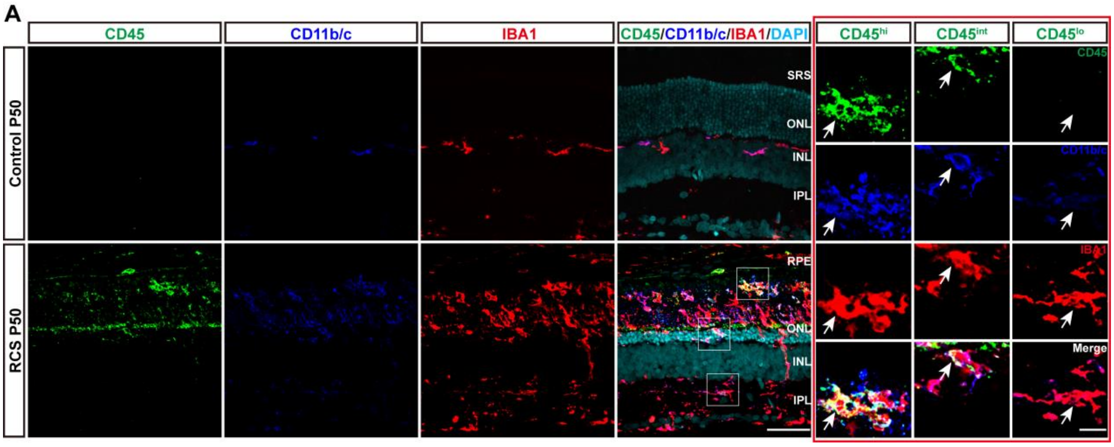

Supplementary Figure 4. A few monocyte-derived macrophages are found in the outer retinas of RCS rats at P50.

(A) Representative high-resolution confocal images showing immunofluorescent staining of CD45, CD11b/c and IBA1 in the retinas of control rats and RCS rats at P50. The right red panel shows the typical classification of $IBA1^{+}$ cells based on CD45 expression in the retinas of RCS rats at a higher magnification.

Abbreviations: RPE, retinal pigment epithelium; SRS, subretinal space; ONL, outer nuclear layer; OPL, outer plexiform layer; IPL, inner plexiform layer; INL, inner nuclear layer; GCL, ganglion cell layer. Scale bars, 50 μm or 10 μm.

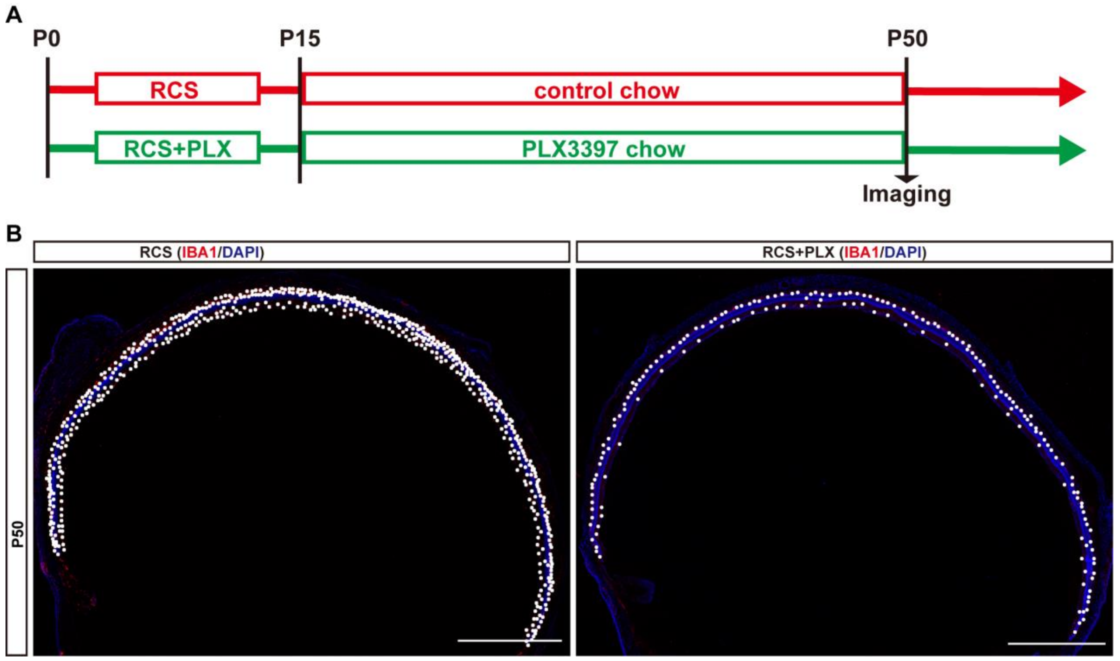

Supplementary Figure 5. Treatment with PLX3397 from P15 to P50 significantly eliminates microglia in the retinas of RCS rats at P50.

(A) Schematic of PLX3397 administration and time points for observation.

(B) Representative panoramic images of retinal sections stained with DAPI (blue) and IBA1 (red) from RCS rats treated with PLX3397 at P50. Each white dot represents a microglial cell. Scale bar, 1 mm (B).

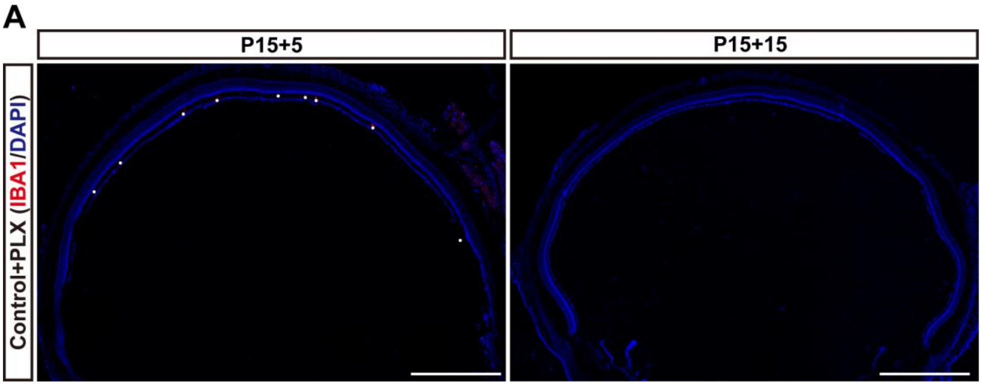

Supplementary Figure 6. Treatment with PLX3397 eliminates nearly all microglia in the retinas of control rats.

(A) Representative panoramic images of retinal sections stained with DAPI (blue) and IBA1 (red) from control rats treated with PLX3397 at P20 and 30. Each white dot represents a microglial cell. Scale bar, 1 mm (A).

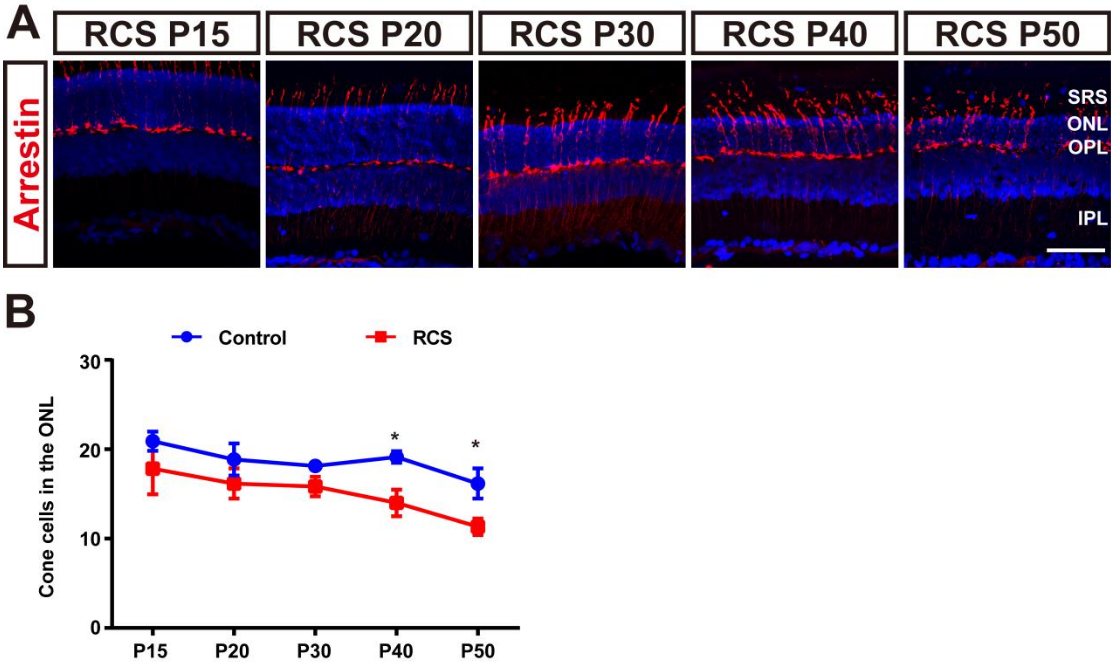

Supplementary Figure 7. Degeneration and loss of cone photoreceptors mainly occurre after P30 in the retinas of RCS rats.

(A) Representative high-resolution confocal images show cone cells (arrestin) in the retinas of RCS rats from P15 to P50.

(B) Quantification of cone cells in the retinas of RCS rats from P15 to P50 (N = 3 eyes from different rats, N = 6-8 images (213 × 213 μm) from each eye).

Abbreviations: SRS, subretinal space; ONL, outer nuclear layer; OPL, outer plexiform layer; IPL, inner plexiform layer. Scale bar, 50 μm (A). Bars represent means; error bars represent SDs. *$p < 0.05$ using a two-way ANOVA (B).

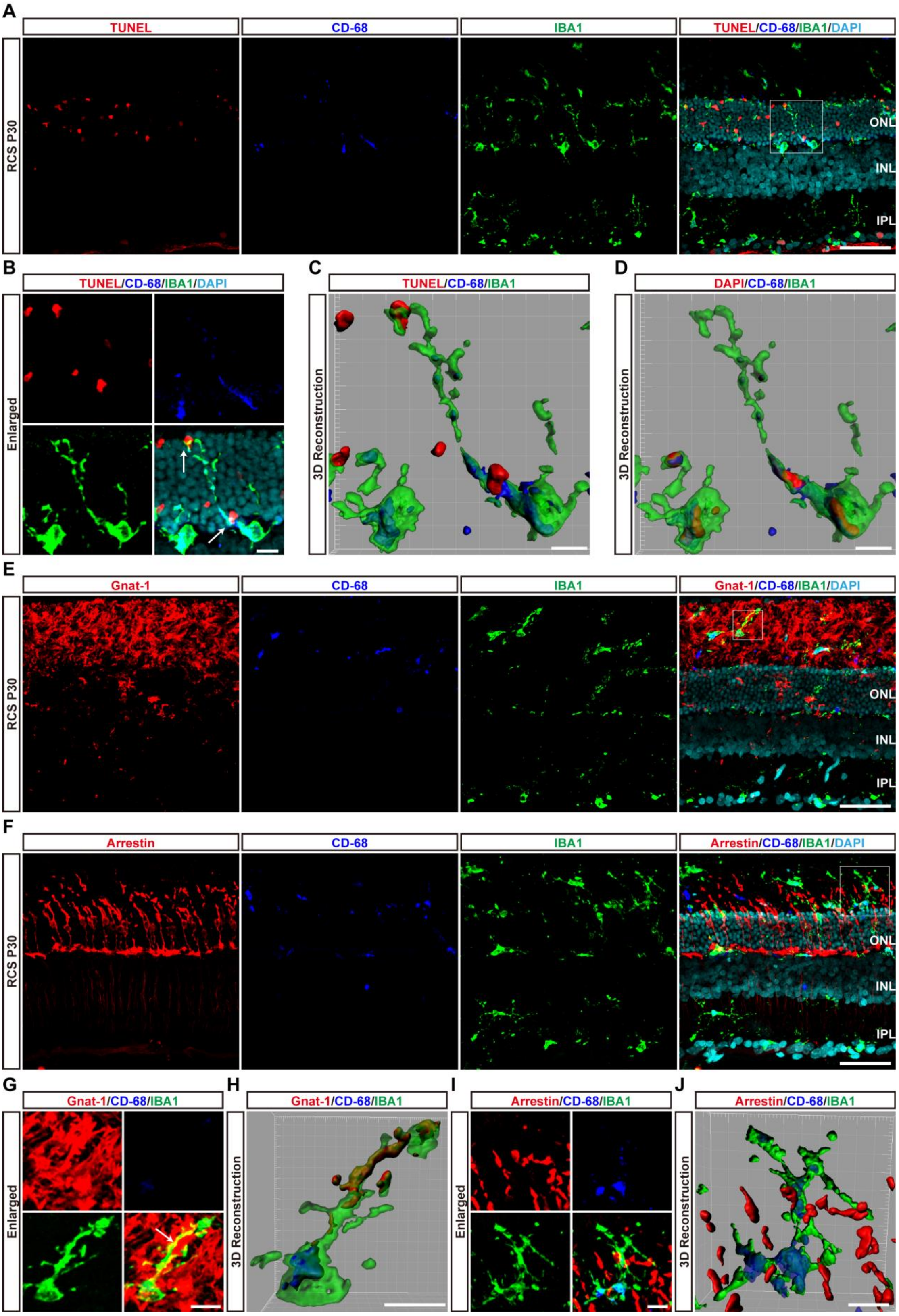

Supplementary Figure 8. Microglia phagocytose apoptotic rods in the photoreceptor layer of RCS rats during the early stage of degeneration.

(A) Representative immunofluorescence images of TUNEL (red), CD-68 (blue), IBA1 (green) and DAPI (cyan) in the retinal sections of RCS rats at P30.

(B) High-resolution confocal images from panel A are shown at a higher magnification.
(C) Three-dimensional reconstruction and surface renderings of microglial cells (green), TUNEL-positive cells (red) and CD-68 (blue) in the retinas of RCS rats at P30.
(D) Three-dimensional reconstruction and surface renderings of IBA1 (green), DAPI (red) and CD-68 (blue) in the retinas of RCS rats at P30.
(E) Representative immunofluorescence images of Gnat-1 (red), CD-68 (blue), IBA1 (green) and DAPI (cyan) in retinal sections from RCS rats at P30.
(F) Representative immunofluorescence images of arrestin (red), CD-68 (blue), IBA1 (green) and DAPI (cyan) in retinal sections from RCS rats at P30.
(G) High-resolution confocal images from panel E are shown at a higher magnification.
(H) Three-dimensional reconstruction and surface renderings of IBA1 (green), Gnat-1 (red) and CD-68 (blue) in the retinas of RCS rats at P30.
(I) High-resolution confocal images from panel F are shown at a higher magnification.
(J) Three-dimensional reconstruction and surface renderings of IBA1 (green), arrestin (red) and CD-68 (blue) in the retinas of RCS rats at P30.
Abbreviations: RPE, retinal pigment epithelial; SS, subretinal space; ONL, outer nuclear layer; OPL, outer plexiform layer; IPL, inner plexiform layer; INL, inner nuclear layer; GCL, ganglion cell layer. Scale bars, 50 μm (A, E and F), 10 μm (B, G and I) or 5 μm (C, D, H and J).

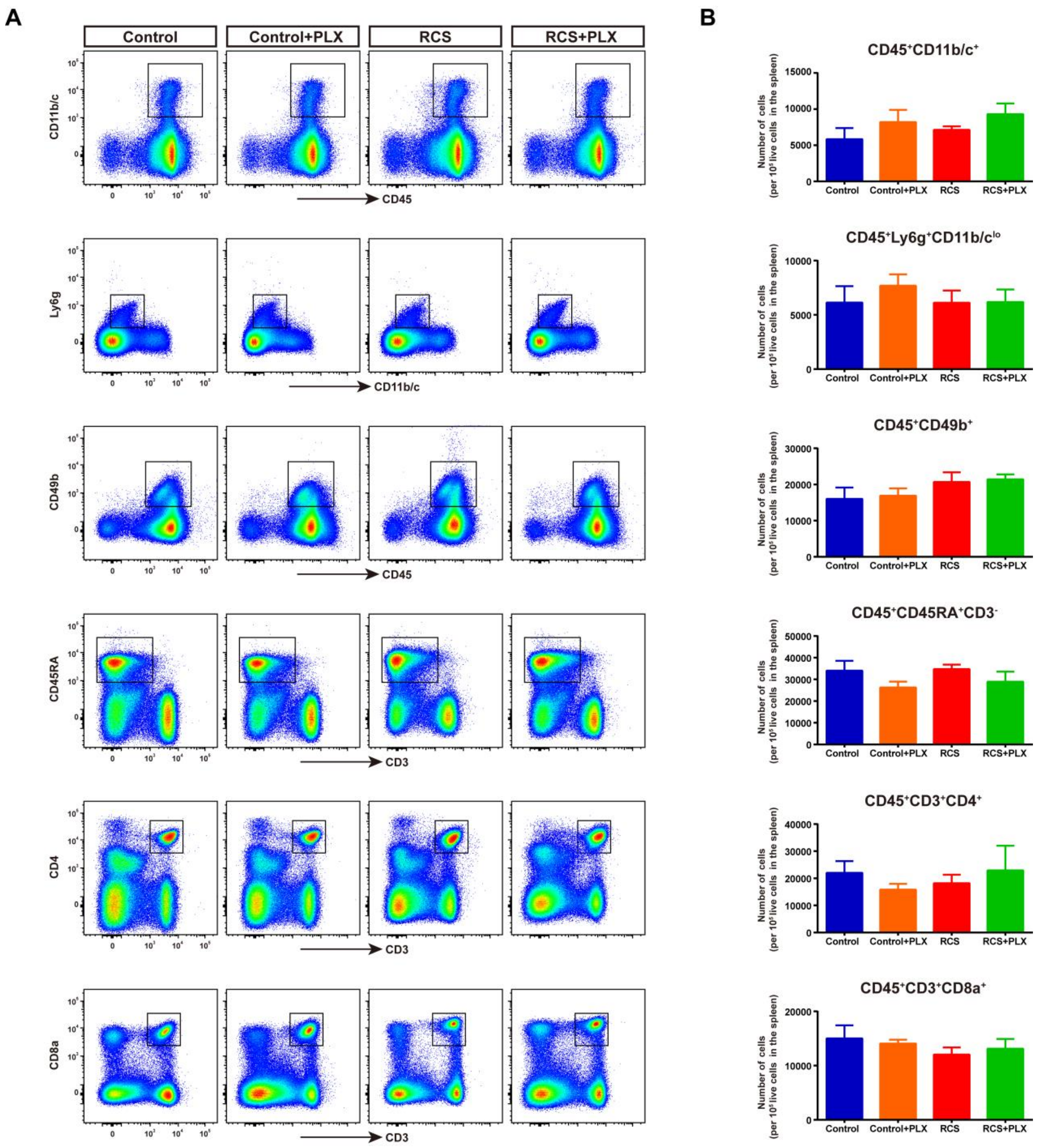

Supplementary Figure 9. Sustained treatment with the CSF1R inhibitor PLX3397 does not influence the number of leukocytes in the spleens of control rats and RCS rats at P50.

(A) Representative flow cytometry plots showing the gating strategy of leukocyte subpopulations isolated from the spleen. Plots show the gating of macrophages (CD45$^+$ CD11b/c$^+$), neutrophils (CD45$^+$Ly6g$^+$CD11b/c^{low}), NK cells (CD45$^+$ CD49b$^+$), B cells (CD45$^+$ CD45RA$^+$CD3$^-$), CD4$^+$ T cells (CD45$^+$ CD3$^+$ CD4$^+$) and CD8$^+$ T cells (CD45$^+$ CD3$^+$ CD8$^+$).

(B) Quantification of retina-infiltrated lymphocytes and neutrophils from control rats (N = 3 rats), control rats treated with PLX3397 (N = 3 rats), RCS rats (N = 3 rats) and RCS rats treated with PLX3397 (N = 3 rats) at P50.

Data represent relative cell numbers in 10^5 living cells per spleen. Bars represent means; error bars represent SDs; two-way ANOVAs were used (B).

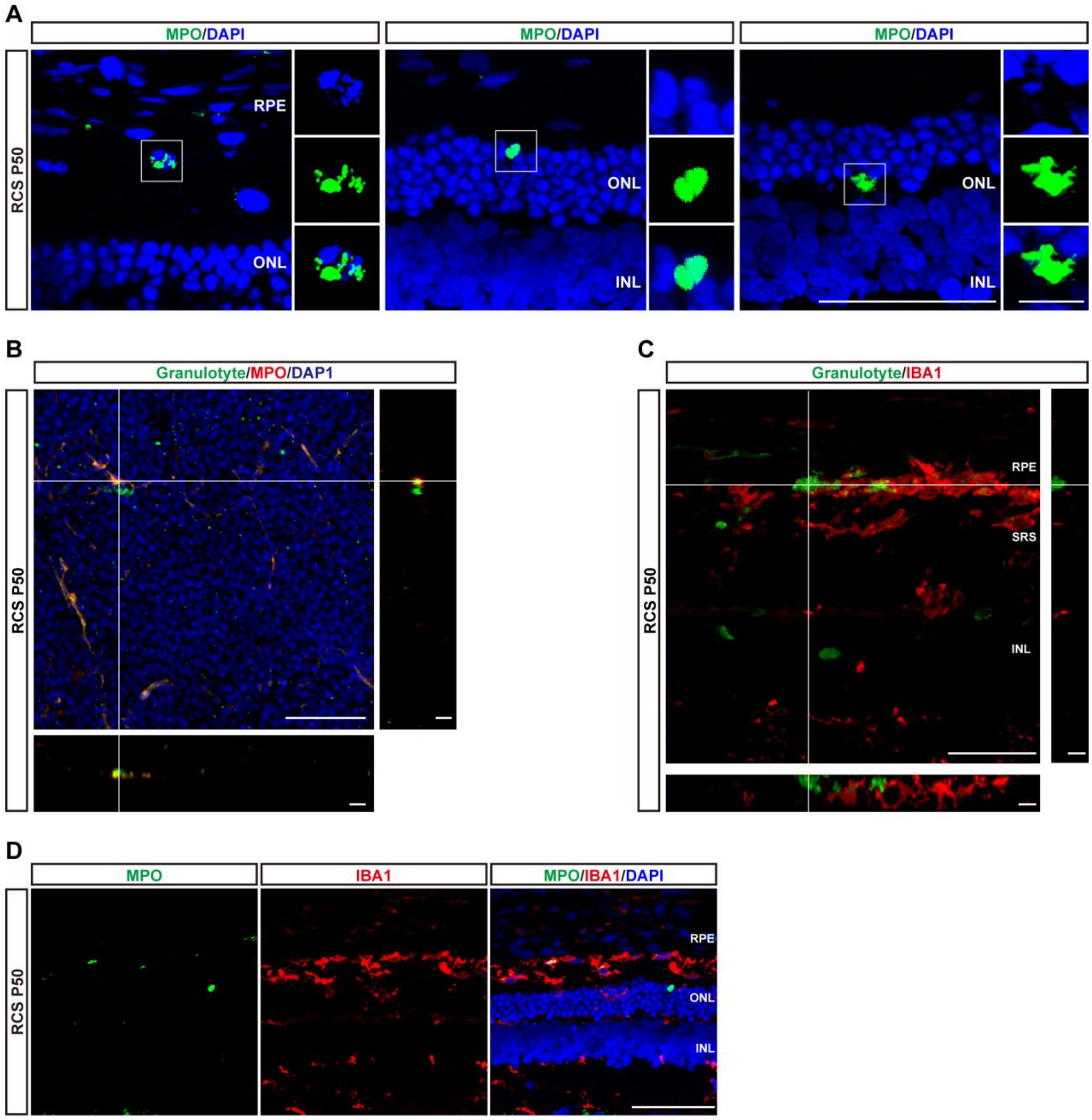

Supplementary Figure 10. Analysis of the neutrophils in the photoreceptor layer of RCS rats at P50.

(A) Representative high-resolution confocal images show neutrophils (MPO) in the outer retinas of RCS rats at P50.

(B) Orthogonal views of high-resolution confocal images showing immunofluorescent staining of His48 and MPO in the flat-mount retinas of RCS rats at P50.

(C) Orthogonal views of high-resolution confocal images showing immunofluorescent staining of His48 and IBA1 in retinal sections of RCS rats at P50.

(D) Representative high-resolution confocal images show the relationship between neutrophils (MPO) and microglia (IBA1) in the retinas of RCS rats at P50. Scale bars, 50 μm (A-D) or 10 μm (A-C).
